# Supplementary material for: HnRNPK maintains single strand RNA through controlling double-strand RNA in mammalian cells
Source: Nat Commun. 2022 Aug 29;13:4865. doi: 10.1038/s41467-022-32537-0 (PMC9424213; doi:10.1038/s41467-022-32537-0)
Supplement: Supplementary file 1 — Supplementary Information [file 41467_2022_32537_MOESM1_ESM.pdf]

Supplementary Figure 1

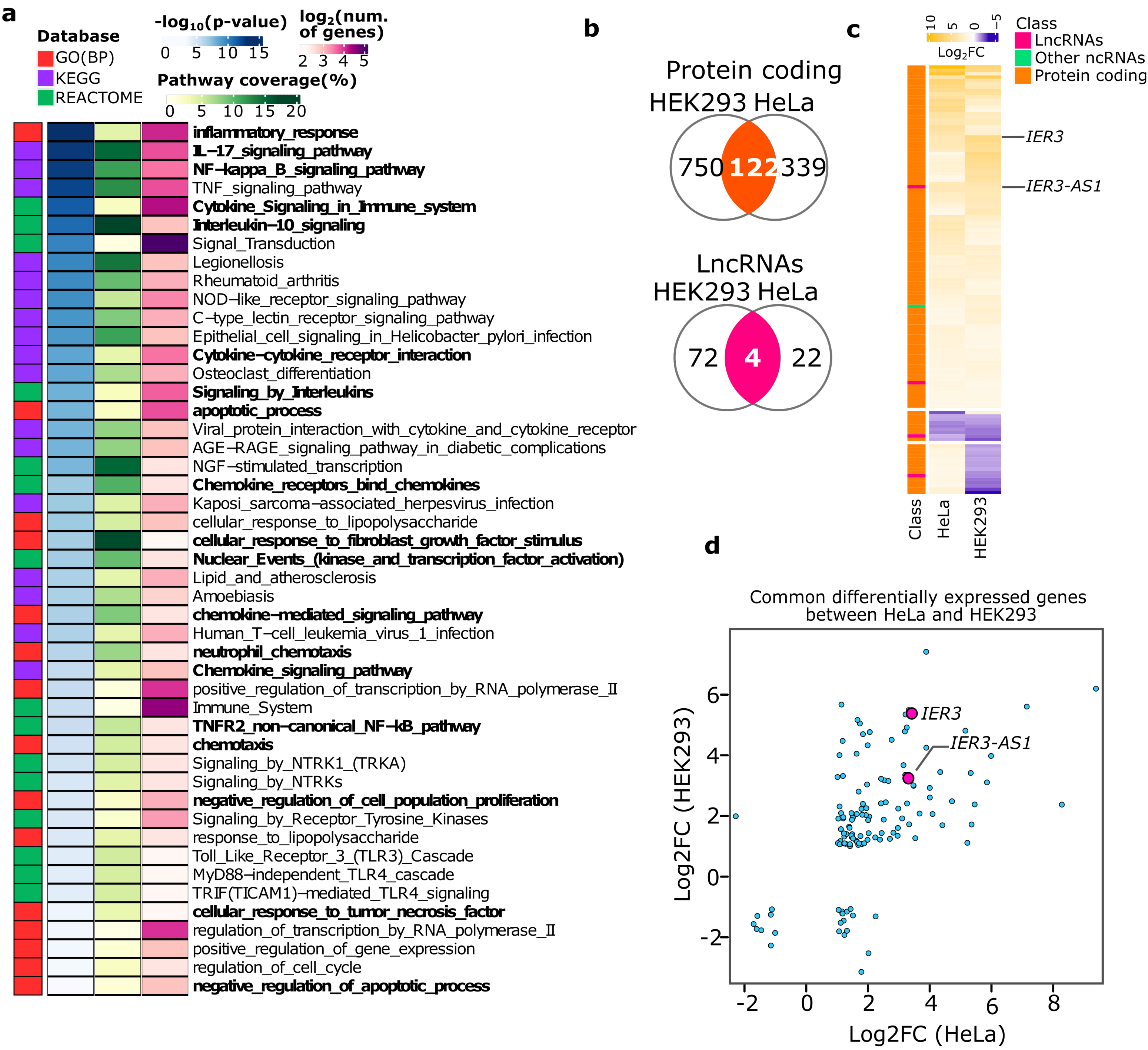

Supplementary Figure 1: a) Heatmaps depict significantly enriched biological functions of FGF-2 induced common protein coding genes from the three cell lines, ranked using geneSCF using databases gene ontology (GO) terms KEGG and REACTOME. b) Venn diagram showing FGF -2 induced commonly upregulated and downregulated DEGs in both HEK293 and HeLa cell lines. c) Heatmap representing the common protein coding genes and non-coding genes showing the same and differential expression pattern in FGF-2 induced HEK293 and HeLa cell lines. d) Log2FC values for the common DEGs in FGF-2 induced HEK293 and HeLa cell lines. Pink dots depict the Log2FC values of IER3 and IER3-AS1.

Supplementary Figure 2

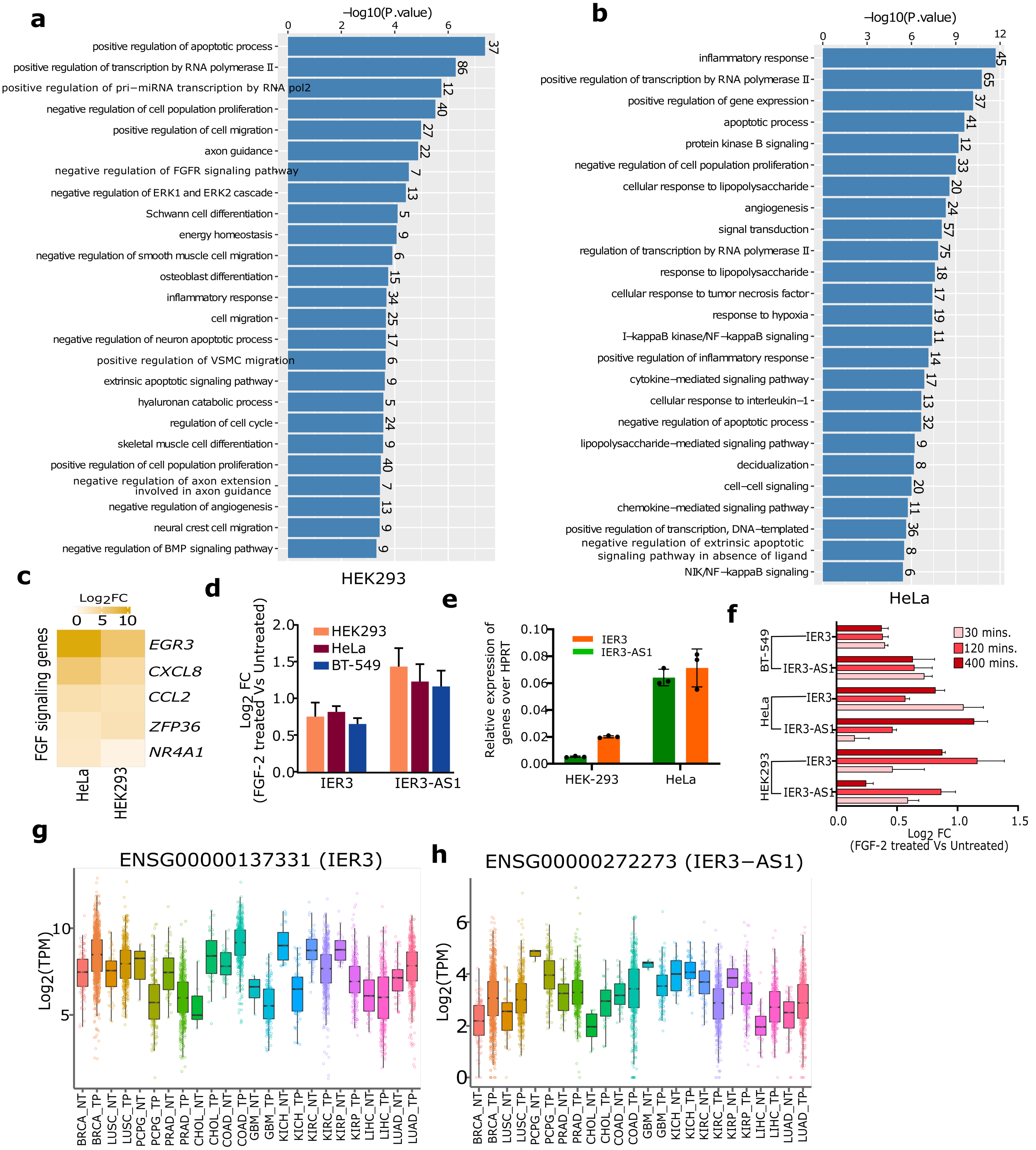

Supplementary Figure 2: a-b) Functional gene enrichment analysis of FGF-2 induced HEK293 (A) and HeLa (B) specific DEGs. c) Heatmap showing the expression pattern of DEGs related to fibroblast growth factor stimulus signaling pathway. d) RT-qPCR validation of FGF-2 dependent induction of IER3 and IER3-AS1 in HEK293, HeLa and BT-549 cell lines. e) Basal expression of IER3 and IER3 -AS1 transcripts in HEK293 and HeLa cell lines. f) Log2FC levels of IER3 and IER3-AS1 at the indicated FGF-2 treatment time points and the data is presented over un-treated samples. Protein lysates from this experiment was used to generate Western blot data presented in Figure 1d. The p values were calculated using two-sided student's t-test and data are presented ± SD from two independent replicates for d-f. g-h) Differential expression status of IER3 (g) and IER3-AS1(h) across different cancer types from TCGA. NT means normal and TP means Tumor. Box plots represent low expression range (lower whiskers), higher expression range, (upper whisker), median, inter quartile range (IQR), and the extreme expression values.

Supplementary Figure 3

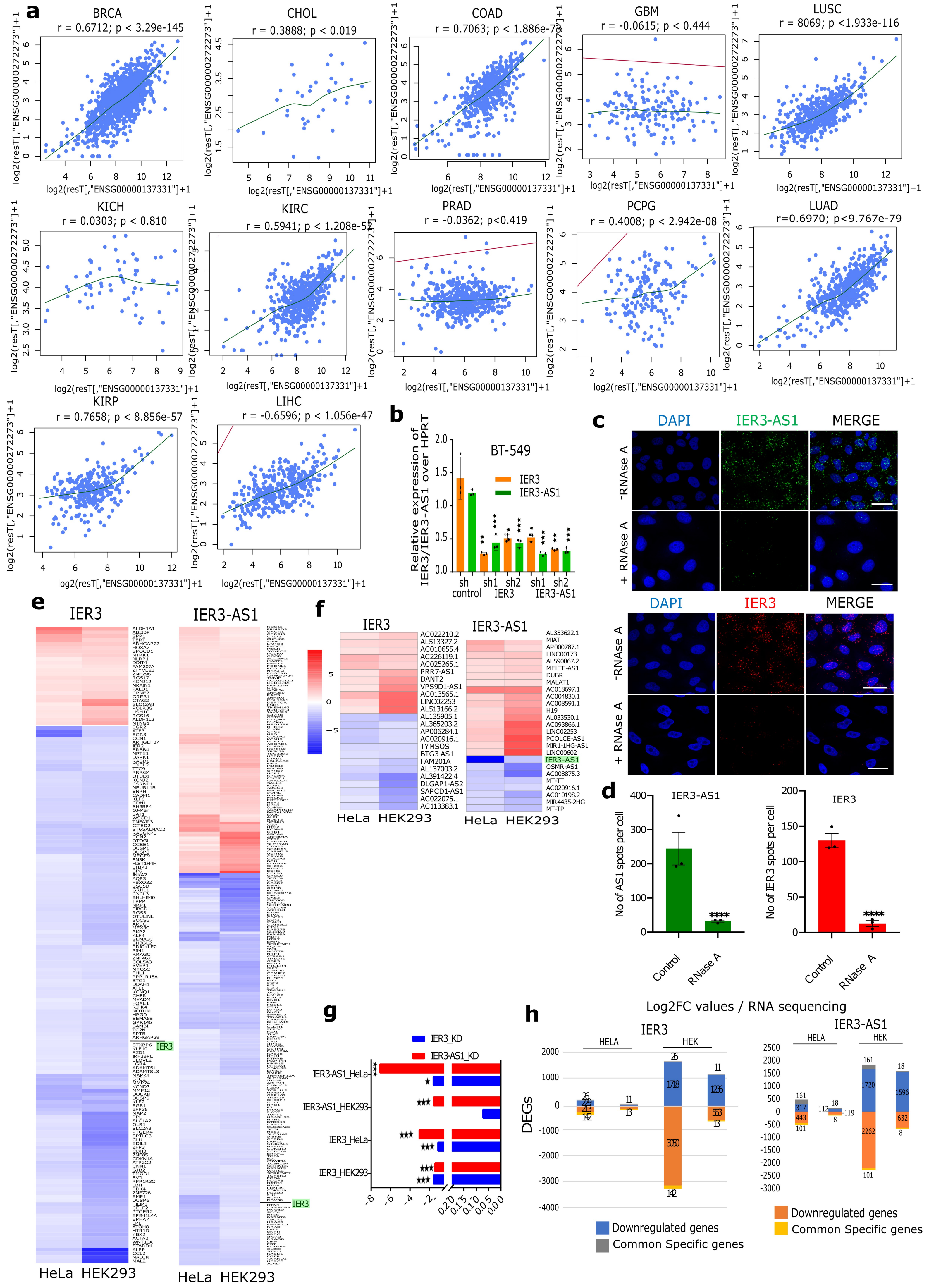

i

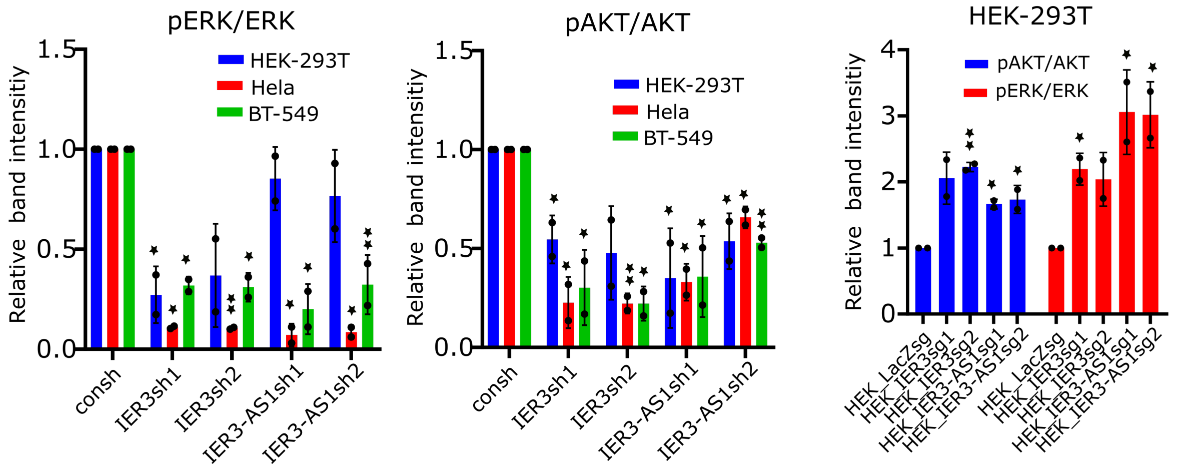

Supplementary Figure 3: a) Expression correlation plots of IER3 and IER3-AS1 in different cancers from TCGA. Correlation co-efficient and p value are mentioned above on each plot. b) RT-qPCR data of BT-549 cells following stable downregulation of IER3 and IER3 -AS1 using lentiviral sh RNAs. The values represent mean ± SD of three independent biological replicates. $*p < 0.05$, $**p < 0.01$ and $***p < 0.001$ by two-sided Student's t test. c-d) RNAscope images of Hela cells showing the significant reduction in the RNA fluorescence signals upon RNaseA treatment. IER3 -AS1 (Green) and IER3 (Red) transcripts in HeLa untreated and RNase A treated cells. DAPI was used to stain nucleus. Indicative scale bar on the images is 50um. d) Graph showing significant reduction in the number of IER3-AS1 (left panel) and IER3 (right panel) RNA signals upon RNaseA treatment quantified as RNA spots per cell using Imaris spot detection tools. A total of untreated cells: n=113, and treated cells: n= 43 for IER3-AS1, and for IER3, untreated: n=74, and treated: n= 36 cells were counted from three independent experiments. Data represents +/ -SEM and p value were calculated by two-sided Student’s t-test. e-f) Heatmaps show the expression (log2FC values) of protein coding (e) and non-coding (f) DEGs in IER3 and IER3 -AS1 KD HEK293 and HeLa cell lines. g) Log2FC values of IER3 and IER3 -AS1 in RNA sequencing data of HEK293 and HeLa cell lines following stable KD of IER3 and IER3-AS1. The stars indicate the adjusted p-value of 0.05 by Benjamini-Hochberg method. $*p < 0.05$, $**p < 0.01$ and $***p < 0.001$. h) Bar graphs showing the number of common and specific DEGs that are upregulated or downregulated in IER3 KD and IER3-AS1 KD HEK293 and HeLa cell lines. i) Histograms showing the relative band intensities from the Western blot (Figure 1 i). The data are presented ± SD from two independent replicates and p values calculated using two-sided Student’s t. $*p \leq 0.05$; $**p \leq 0.005$; $***p \leq 0.0005$.

Supplementary Figure 4

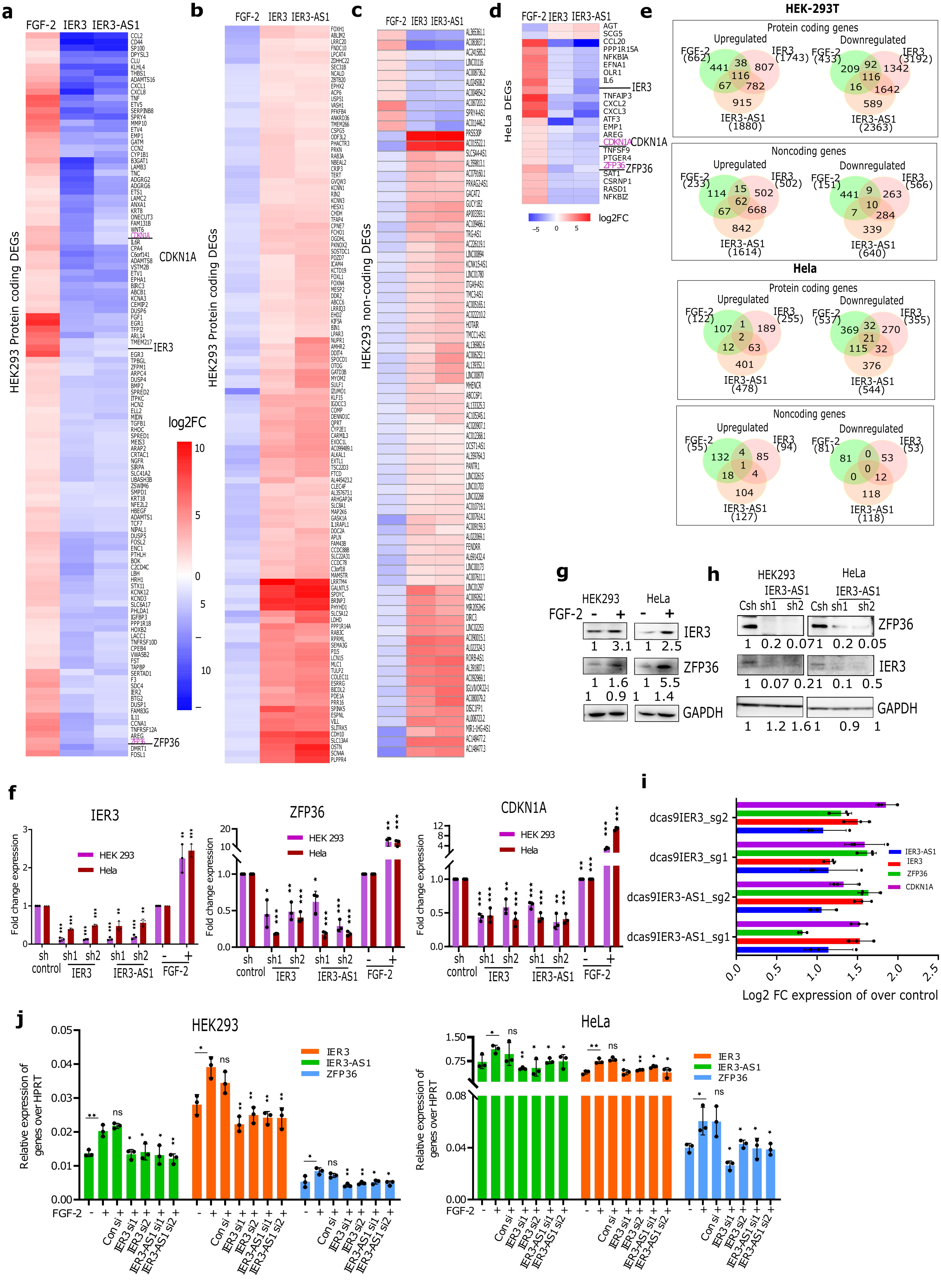

Supplementary Figure 4: a-b) Heatmaps showing the log2FC values of FGF-2 upregulated (a) and downregulated (b) protein coding genes in IER3-AS1 or IER3 KD HEK293 cells. c) Heatmaps showing the Log2FC values of FGF-2 upregulated and downregulated noncoding genes in IER3-AS1 or IER3 KD HEK293 cells. d) Heatmaps showing the Log2FC values of FGF-2 upregulated and downregulated protein coding genes in IER3-AS1 or IER3 KD HeLa cells. The bar scale shows the log2FC values with the indicated colors in the heatmaps. e) Venn diagrams show the overlap of FGF-2 regulated protein coding genes (upper panel) and non-coding genes (lower panel) with DEGs from IER3 or IER3-AS1 KD HeLa and HEK293 cells. f) Bar plots show RT-qPCR analysis of IER3 and its target genes in IER3 and IER3-AS1 stable KD or FGF-2 treatment in HeLa and HEK293 cell lines. g-h) Western blot analysis of IER3 and its indicated target genes upon FGF-2 treatment (g) or IER3 and IER3-AS1 KD (h) in HeLa and HEK293 cells. GAPDH was a loading control. The band intensities were quantified using ImageJ software and the values are mentioned below each band. Same results were observed in the independent biological replicate. i) RT-qPCR analysis showing log2FC expression levels of IER3 and its indicated target genes in HEK293 cells following activation of IER3 or IER3-AS1 using CRISPR/dCas9 activation system using IER3 and IER3-AS1 specific guide RNAs. LacZ guide RNA was used as a control. j) Bar graphs show RT-qPCR analysis of IER3 and its indicated target genes in HEK293 and HeLa cells treated with FGF-2 followed by IER3 or IER3-AS1 KD. The values represent mean ± SD of three independent biological replicates. $*p < 0.05$, $**p < 0.01$ and $***p < 0.001$ by two-sided Student's t test for f, i and j.

Supplementary Figure 5

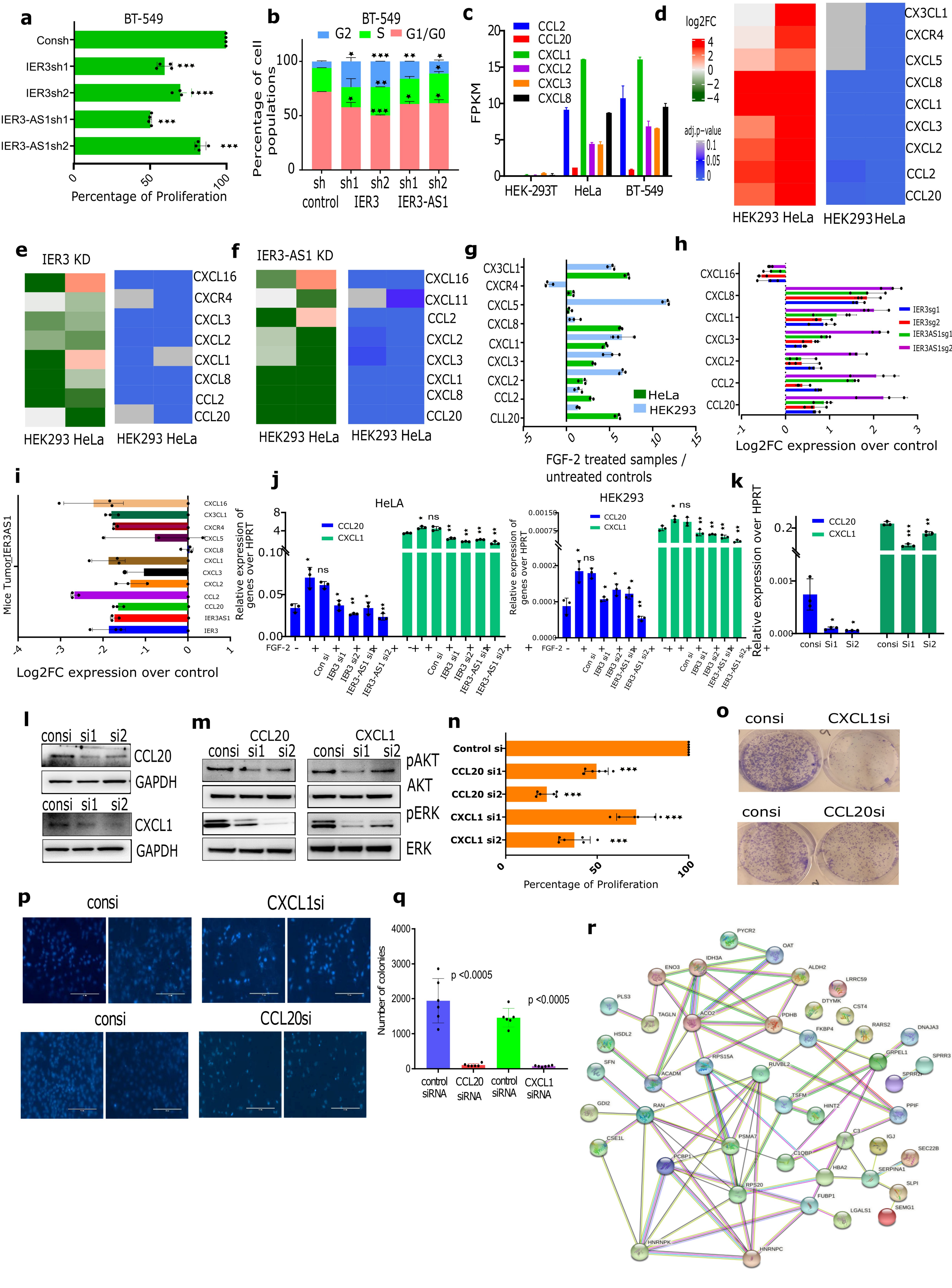

Supplementary Figure 5: a) Cell viability assay showing the percentage of proliferation for IER3 and IER3 -AS1 stable KD BT-549 cells. b) Distribution plots showing the percentage of cell populations of G0/G1, S and G2 phases of cell cycle in BT -549 cells. c) Basal expression levels of chemokines in the RNA sequencing data from HEK293, HeLa and BT -549 cell lines. d-f) Heatmaps showing the log2FC values of chemokines that were significantly upregulated by FGF-2 (d) and downregulated in IER3 KD (e) and IER3-AS1 KD (f) HeLa cells. The blue colored Heatmaps indicates p-value. The bars to the left side of the heatmaps show color code for upregul ation, down-regulation and p value. g-i) RT-qPCR validation of chemokine expression levels in FGF2 treated HeLa and HEK293 cells (g), CRISPR/dcas9 dependent activation of IER3 or IER3-AS1 in HEK293 cells (h), in xenografts (n =3) lacking IER3-AS1 (i). The bars represent the Log2FC chemokine expression values over controls (n= 2 independent biological replicates). j) Expression levels of chemokines in HEK293 and HeLa cells treated with FGF-2 followed by IER3/IER3-AS1 KD using siRNAs. k) Expression values of chemokines in the indicated siRNAs treated samples, presented in relation to control siRNAs. l) Western blots showing the protein levels following their downregulation using siRNAs. m) Western blot analysis of protein levels in CCL20 and CXCL1 downregulated HeLa cells. n) Cell viability assay showing the percentage of cell proliferation of HeLa cells following CCL20 and CXCL downregulation. o) Colony forming efficiency of control and chemokine (CXCL1 and CCL20) KD HeLa cells. Similar results were obtained in the independent biological replicates for m-o. p) Invasion assay of HeLa cells depleted with CXCL1 and CCL20. q) Bar graphs showing quantification of the invasion assay in Supplementary Figure 5p (n= 2 independent biological replicates). r) Protein-protein interaction results obtained from STRING database for the top IER3-AS1 interacting proteins from ChOP-mass spectrometry data. For figures a, b, j, k and n. The values represent mean ± SD of two biological experiments. $*p < 0.05$, $**p < 0.01$ and $***p < 0.001$ by two-sided Student's t-test.

Supplementary Figure S6

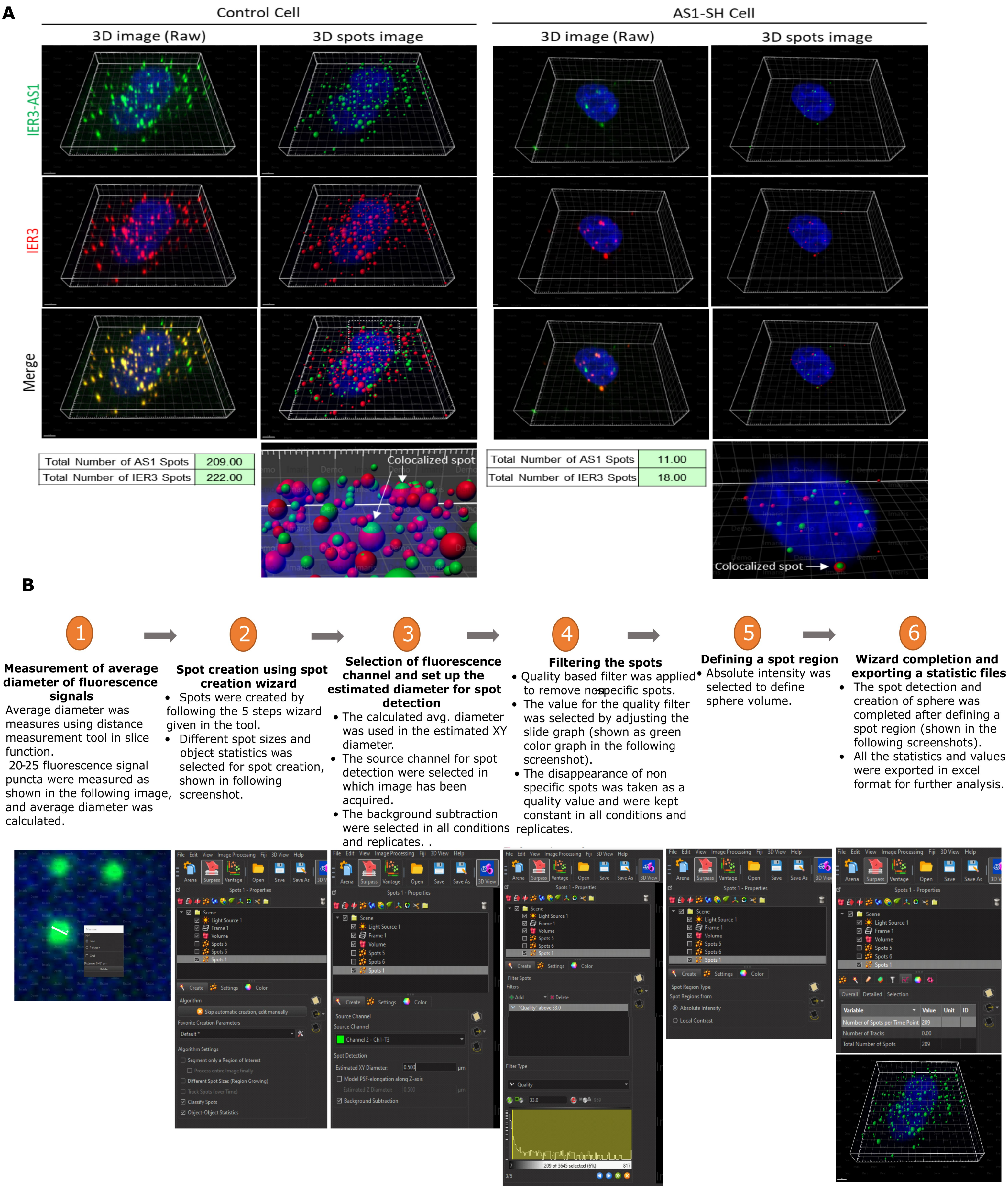

Supplementary Figure S6: A) RNAscope images of the 3D reconstructed cell (cropped raw confocal image) using Imaris(9.8.2 version) showing the IER3 (red) and IER3-AS1 (green) RNA signals in Control sh HeLa cells (left side of left panel) and IER3-AS1sh HeLa cells (left side of the right panel). Right side of the left panel, and right side of right panel showing detected spots using spot detection tool on a 3D reconstructed image: green sphere represents IER3-AS1 and red sphere represents IER3. Magnified area of co-localized spots was shown below the merged image depicted by white doted box. Number of total spots detected by the spot detection tool were given below each respective panel and co-localized spots were shown by white arrows in the magnified image. B) Schematic workflow for the spot detection tool.

Supplementary Figure 7

a

FIMO Analysis (Find Individual Motif Occurence)

Sequence Name	Strand	Start	End	p-value	q-value	Matched Sequence
IER3-AS1	+	9	13	0.000709	0.198	CCTCC
IER3	+	91	95	0.000709	0.198	CCTCC
IER3-AS1	-	337	341	0.000709	0.198	CCTCC
IER3-AS1	+	351	355	0.000709	0.198	CCTCC
IER3	+	357	361	0.000709	0.198	CCTCC
IER3-AS1	+	399	403	0.000709	0.198	CCTCC
IER3-AS1	+	415	419	0.000709	0.198	CCTCC
IER3	+	473	477	0.000709	0.198	CCTCC
IER3	+	789	793	0.000709	0.198	CCTCC
IER3	-	952	956	0.000709	0.198	CCTCC
IER3	-	968	972	0.000709	0.198	CCTCC
IER3	-	1016	1020	0.000709	0.198	CCTCC
IER3	+	1030	1034	0.000709	0.198	CCTCC

b

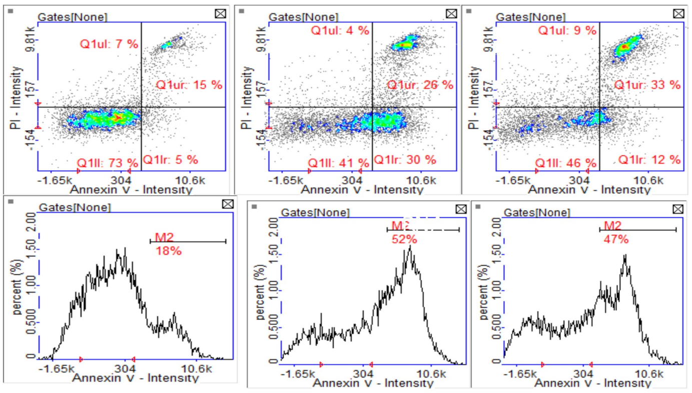

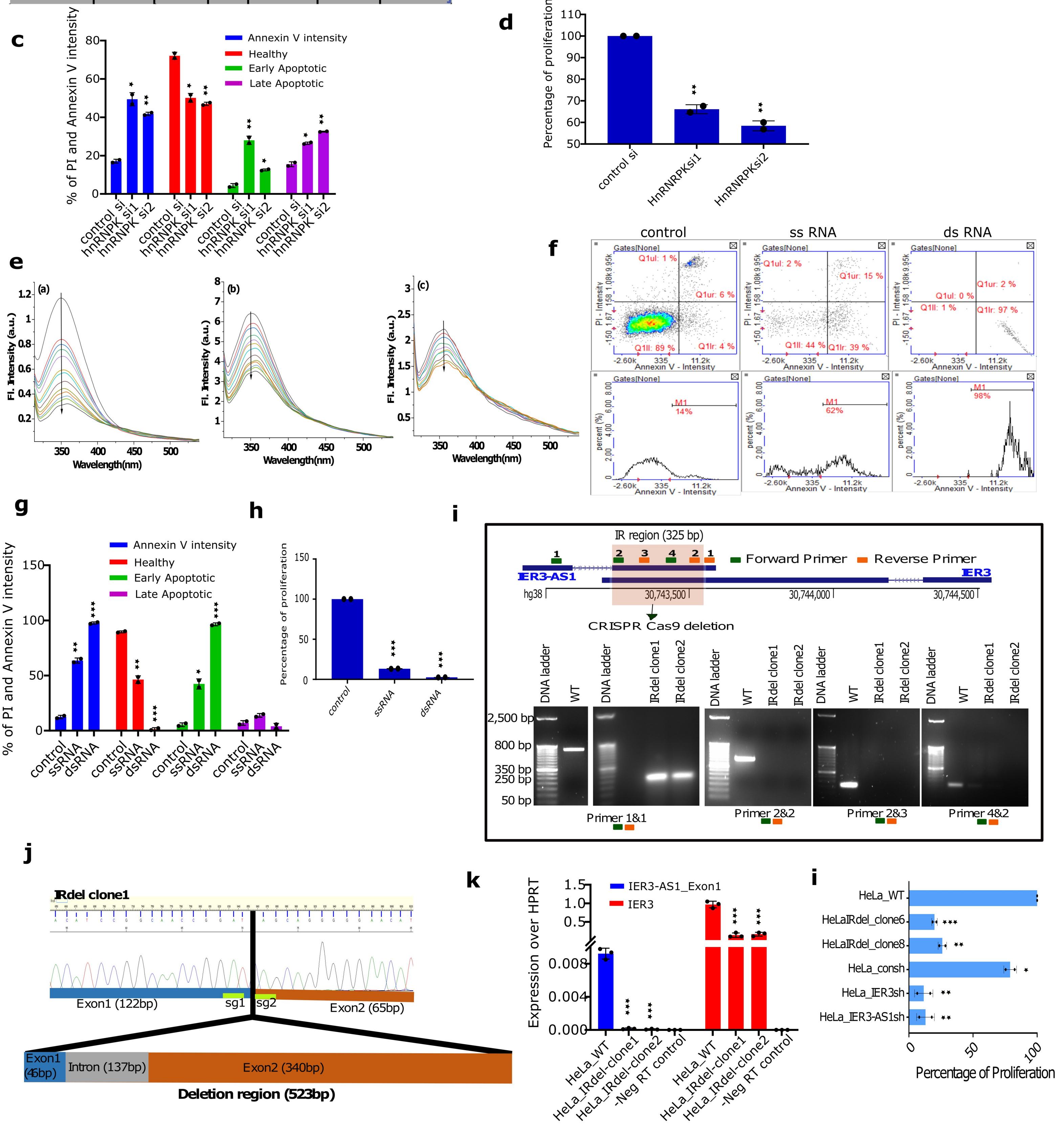

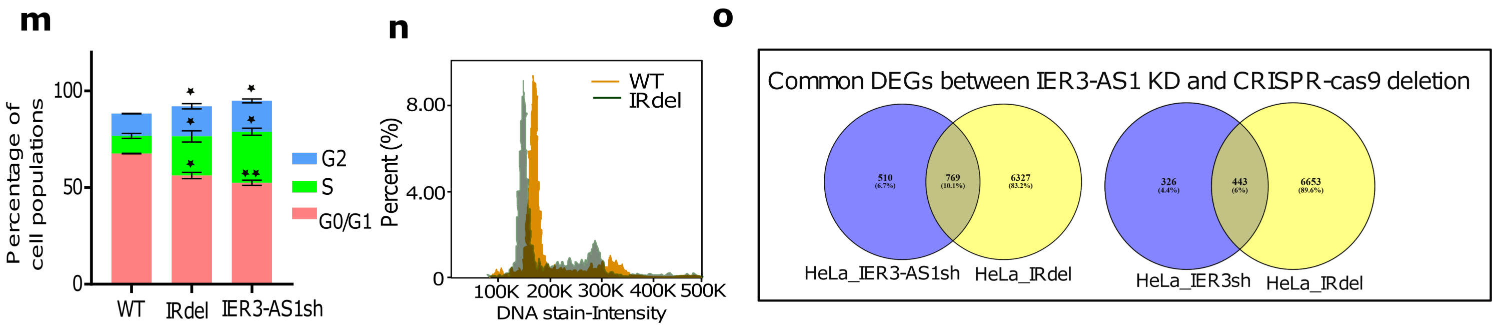

Supplementary Figure 7: a) Table showing the number and location of CCTCCC SIRLOIN motifs analyzed using FIMO. b and f) Scatter plots and histograms of Annexin V positive HeLa cells for control and hnRNPK si RNA samples (b) and *in vitro* transcribed ssRNA (IR region in IER3 -AS1 orientation) and annealed IER3/IER3-AS1 dsRNA (IR region transcribed in both IER3 and IER3-AS1 orientations) (f). Cells were stained with Annexin V - Fluorescein Isothiocyanate conjugated antibody and propidium iodide and analyzed using NucleoCounter NC - 3000. In the right two quadrants, ur and lr show the percentage of Annexin V and PI positive cells. c and g) The bar plots showing the Annexin V intensities based on the scatter plots and histograms shown in Supplementary Fig 7b and 7f, respectively. d and h) Cell proliferation potential of HeLa cells following hnRNPK KD(d) and ssRNA/dsRNA transfected HeLa cells(h). e) Tryptophan fluorescence spectra of the H nRNPK protein with increasing amount of IER3, IER3-AS1 and IER3/IER3-AS1 dsRNA. i) The schematic representation of IR and the location of the primer sequences used for checking the CRISPR targeted deletion of IR region. Below the schematic is agarose gel showing the PCR amplification of the IR region for wild-type and CRISPR delet ed clones using different combinations of the primer sets as indicated in the schematic diagram. j) Sanger sequencing data for the HeLa CRISPR deleted IR clone showing the exact location and the size of the deleted region. K) Expression of IER3 - AS1 and IER3 in control HeLa cells and HeLa IR CRISPR deleted clones. l) Cell viability assay showing the percentage of proliferation for the IRdel stable cell clones compared to the IER3 -AS1sh and IER3sh stable knockdown cells. m-n) showing distribution plots of different cell cycle phases (m) and histograms showing shift in the S phase (n) in control HeLa and HeLa-IRdel cell clones. o) Venn diagrams showing the overlap of common DEG from RNA-sequencing data. For figures c, d, g, h, k, l and m the values represent mean ± SD of two (figure k, n =3) biological experiments. $*p < 0.05$, $**p < 0.01$ and $***p < 0.001$ by two-sided Student's t test.

Supplementary Figure 8

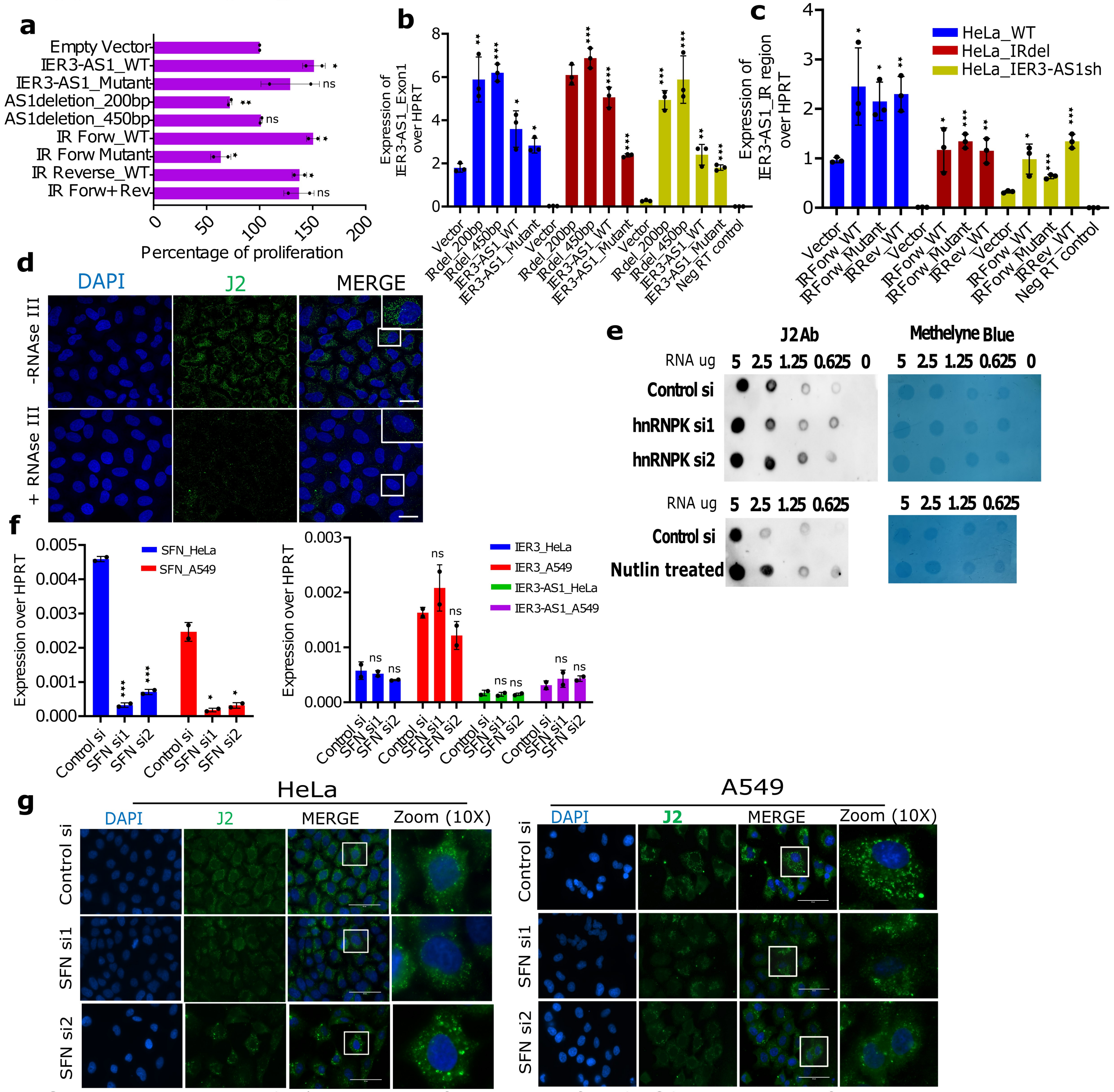

Supplementary Figure 8: a) Percentage of cell viability of IER3-AS1sh cell line transfected with various plasmids containing wild type, IER3-AS1 deletion and IER3-AS1 mutant clones (CCTCC motifs mutated to AAGAA). b and c) Expression levels of IER3-AS1 exon1 (b) and IR region (c) from total RNA obtained from HeLa_WT, HeLa_IRdel and HeLa_IER3-AS1sh cell lines transfected with various wild-type, IER3-AS1 deletions and IER3-AS1 SIRLOIN mutant. d) Immunofluorescence Images of HeLa cells showing decrease in J2-dsRNA specific signals (green) in RNase III treated HeLa cells. The panels to the right show merged images. Magnified J2 signals (puncta) were shown in an inset at the upper right corner indicated by white box. DAPI staining was used to locate the nucleus. Indicative scale bar on the images is 50um. e) Dot blots showing double strand RNA (dsRNA) levels in HeLa cells treated with control si or hnRNPKsi. Total RNA from control siRNA or hnRNPK siRNA treated cells was loaded onto a nylon membrane in a dilution series of 5000, 2500, 1250 and 625 ng per dot. Membranes were then probed with dsRNA-specific J2 antibody. The lower panel shows methylene blue staining of the blot from the upper panel to control for the loading of the indicated RNA amounts. HeLa cells treated with Nutlin-3a RNA samples were shown as a positive control for J2 antibody. f) Expression levels of SFN, IER3 and IER3-AS1 in control and SFN downregulated HEK293 and HeLa cells. SFN was downregulated using siRNAs. g) Representative IF images showing J2 staining (green) following SFN siRNA and control siRNA treatment of HeLa cells. DAP1 staining was used to locate the nucleus. The panel to extreme right show the magnified images (10X zoom) highlighting the areas marked in white color boxes from the merged panel. Figures d, e and g were performed in two biological replicates and similar results were obtained. For the figures, a, b, c and f the values represent mean ± SD of three biological experiments. $*p < 0.05$, $**p < 0.01$ and $***p < 0.001$ by two-sided Student's t test.

Supplementary Table 1 – List of RT-PCR primers used in this study

Gene	Primer sequences (5'-3')
IER3	5' TCTTCACCTTCGACCCTCTC 3'(Forward primer)
	5' ACACCCTCTTCAGCCATCAG 3' (Reverse primer)
IER3-AS1	5' GGATGCTGGGTTCTGTGACT 3'(Forward primer)
	5' GCGGACCATTAGGAATGAGA 3' (Reverse primer)
HPRT	5' GCTATAAATTCTTTGCTGACCTG 3'(Forward primer)
	5' AATTACTTTTATGTCCCCTGTTGACTGG 3' (Reverse primer)
GAPDH	5' TTAAAAGCAGCCCTGGTGAC 3'(Forward primer)
	5'CTCTGCTCCTCCTGTTCGAC 3' (Reverse primer)
ZFP36	5' ACTTCAGCGCTCCCACTCT 3'(Forward primer)
	5' GACTCAGTCCCTCCATGGTC 3' (Reverse primer)
CDKN1A	5' GGAAGACCATGTGGACCTGT 3'(Forward primer)
	5' GGATTAGGGCTTCCTCTTGG 3' (Reverse primer)
AL121603	5' CCGGCTCTTACTCGTAGTGG 3'(Forward primer)
	5' ACCTTACCGGCTTTCCTTTC 3'(Reverse primer)
AC005077	5' CAGCTGTGGCCTCACATTG 3'(Forward primer)
	5' ATCGGGACACAGGAGCTG 3' (Reverse primer)
AL357060	5' AACCTCTGCAGCAGTGACCT 3'(Forward primer)
	5' CAGCCCAAACTTTTCAGAGC 3' (Reverse primer)
AC145422	5' GCAAGGGGAGCAGATAGATG 3'(Forward primer)
	5' GGATGAGAGCCAACGTCTTC 3' (Reverse primer)
TDH	5' GCTCCAACTCTGGGTTCGTA 3'(Forward primer)
	5' CTGTGGCCTCTACTCACTCG 3' (Reverse primer)
STPG3-AS1	5' GGAGGCCATGGTTCCACT 3'(Forward primer)
	5' CCTGGCTCAGCACCTGTAAG 3' (Reverse primer)
P65	5' GGCATCCGTCGACAACTCCG 3'(Forward primer)
	5' CCTGAAAGGAGGCCATTGGGG 3' (Reverse primer)
CCL20	5' CAGCACTCCCAAAGAACTGG 3'(Forward primer)
	5' CGTGTGAAGCCCACAATAAA 3' (Reverse primer)
CCL2	5' GCCTCCAGCATGAAAGTCTC 3'(Forward primer)
	5' CAGATCTCCTTGGCCACAAT 3' (Reverse primer)
CXCL2	5' CTCAAGAATGGGCAGAAAGC 3'(Forward primer)
	5' AAACACATTAGGCGCAATCC 3' (Reverse primer)
CXCL3	5' CCCCATGGTTCAGAAAATCA 3'(Forward primer)
	5' CCTTTCCAGCTGTCCCTAGA 3' (Reverse primer)
CXCL16	5' GAAAGCTTGCCTCAATCCTG 3'(Forward primer)
	5' TCCTAAGCGATGCTCAAACA 3' (Reverse primer)
CXCL8	5' AAGAAACCACCGGAAGGAAC 3'(Forward primer)
	5' AAATTTGGGGTGGAAAGGTT 3' (Reverse primer)
CXCL5	5' CCACTATGAGCCTCCTGTCC 3'(Forward primer)
	5' CTATGGCGAACACTTGCAGA 3' (Reverse primer)
CXCR4	5' CAGCAGGTAGCAAAGTGACG 3'(Forward primer)
	5' GTAGATGGTGGGCAGGAAGA 3' (Reverse primer)

CX3CL1	5’ GGCTCCGATATCTCTGTCGT 3’(Forward primer)
	5’ CATGATGCCTGGTTCTGTTG 3’ (Reverse primer)
CXCL16	5’ CCATGGGTTCAGGAATTGAT 3’(Forward primer)
	5’ CAAGGTGGACAGGAGCATCT 3’ (Reverse primer)
hnRNPK	5’ AGCACTGCAGACGCCATTAT 3’(Forward primer)
	5’ TCCTTGCAGAGCAGAACTGA 3’ (Reverse primer)
hnRNPC	5’ CAGCAGCCAGGATGACCT 3’(Forward primer)
	5’ CCCAATGAATACACGGGAGT 3’ (Reverse primer)
NEAT1	5’ CAGTTAGTTTATCAGTTCTCCCATCCA 3’(Forward primer)
	5’ GTTGTTGTCGTCACCTTTCAACTCT 3’ (Reverse primer)
ERVL	5’ATATCCTGCCTGGATGGGGT 3’(Forward primer)
	5’ GAGCTTCTTAGTCCTCCTGTGT 3’ (Reverse primer)
MTL2B4	5’ GGAGAAGCTGATGGTGCAGA 3’(Forward primer)
	5’ ACCAACCTTCCCAAGCAAGA 3’ (Reverse primer)
IER3-AS1_IR	5’ GCGGCGGGTACCCCAGGGAGAACATACTAGGCGATC 3’(Forward primer)
	5’ GCGGCGGGATCCCTCTTGGTATTTATTGAGCTTTGT 3’ (Reverse primer)
IER3_IR	5’GCGGCGGGTACCAGGTCTCTTGGTATTTATTGAGCTTT 3’(Forward primer)
	5’GCGGCGGGATCCAGGGAGAACATACTAGGCGATCTCGAC 3’(Forward primer)

Supplementary Table 2 – List of Primers used for checking CRISPR-cas9 IR deletion and amplifying wild type and mutant sequences

Primers	Primer sequences (5’-3’)
IER3-AS1IR_Upst_Dnst	5’ CGTAAGGGTGATTGCCACAT 3’(Forward primer)
	5’ CGTCCTCCTAGGTGATGGAG 3’ (Reverse primer)
IER3-AS1_IR_5' region	5’ ATGCTGGGTTCTGTGACTCC 3’(Forward primer)
	5’ CGACTGTCGAGATCGCCTA 3’ (Reverse primer)
IER3-AS1_IR_3' region	5’ CGCCGAAGTCTCACACAGTA 3’(Forward primer)
	5’ ACTGCGGCAAAGTAGGAGAA 3’ (Reverse primer)
IER3-AS1_200bp	5’ TAAGCAAAGCTTGGGTGAGGGACTTGACCTC 3’(Forward primer)
	5’ TGCTTAGCGGCCGCACGGAGCGACTGTCGAGAT 3’ (Reverse primer)
IER3-AS1_450bp	5’ TAAGCAAAGCTTGGGTGAGGGACTTGACCTC 3’(Forward primer)
	5’ TGCTTAGCGGCCGCTGGAACTGCGGCAAAGTAG 3’ (Reverse primer)
IER3-AS1_Full length	5’ TAAGCAAAGCTTGGGTGAGGGACTTGACCTC 3’(Forward primer)
	5’ TGCTTAGCGGCCGCCGTCCTCCTAGGTGATGGAG 3’ (Reverse primer)

IER3-AS1_IR forw	5’ GCGGCGGGTACCCCAGGGAGAACATACTAGGCGATC 3’(Forward primer)
	5’ GCGGCGGGATCCCTCTTGGTATTTATTGAGCTTTGT 3’ (Reverse primer)
IER3-AS1_IR Rev	5’ GCGGCGGGTACCAGGTCTCTTGGTATTTATTGAGCTTT 3’(Forward primer)
	5’ GCGGCGGGATCCAGGGAGAACATACTAGGCGATCTCGAC 3’ (Reverse primer)

Supplementary Table 3 – List of shRNAs, sgRNAs, siRNAs and Oligos used for ChIRP assay

**shRNAs target sequence**		
**Name**	**Company**	**Target Sequence**
IER3_Oligo2_S	Sigma Aldrich	GGGCTCCGAAGTCAGATTAAA
IER3_Oligo2_AS	Sigma Aldrich	GGGCTCCGAAGTCAGATTAAA
IER3_Oligo3_S	Sigma Aldrich	GGTACGCCTGGTGTTTCTTTG
IER3_Oligo3_AS	Sigma Aldrich	GGTACGCCTGGTGTTTCTTTG
IER3AS1_Oligo2_S	Sigma Aldrich	TGATTGCCACATCTCGGATTC
IER3AS1_Oligo2_AS	Sigma Aldrich	TGATTGCCACATCTCGGATTC
IER3AS1_Oligo3_S	Sigma Aldrich	ACCGCAGACTGGGCAATGAAA
IER3AS1_Oligo3_AS	Sigma Aldrich	ACCGCAGACTGGGCAATGAAA
Control shRNA particle from Sigma Aldrich		
**sgRNAs target sequence**		
**Name**	**Company**	**Target Sequence**
IER3-AS1sg1	Sigma Aldrich	TCGCGTCTGCCGCAGCAACG
IER3-AS1sg2	Sigma Aldrich	CTCACCTTCCGGGACGCGGG
IER3-AS1sg3	Sigma Aldrich	GGGAAGGGCGGGGTCGCGCA
IER3sg1	Sigma Aldrich	AAATTCCGACGATTAAACAA
IER3sg2	Sigma Aldrich	ACACACTCACAACGTGCAGT
IER3sg3	Sigma Aldrich	CAGGCACATGTCGAGGCATG
**3′-biotin-TEG modified oligos for modified ChIRP-mass spec**		
**Name**	**Company**	**Target Sequence**
IER3-AS1_Probe1	Sigma Aldrich	aatccgagatgtggcaatca
IER3-AS1_Probe2	Sigma Aldrich	ttttcctggagtcacagaac
IER3-AS1_Probe3	Sigma Aldrich	gaatgagatccgtgagatcc
IER3-AS1_Probe4	Sigma Aldrich	ggtcgtaagtttaggaggtg
IER3-AS1_Probe5	Sigma Aldrich	aatgcaggtctcttggtatt
IER3-AS1_Probe6	Sigma Aldrich	ggttttcccaggtagttgcc
IER3-AS1_Probe7	Sigma Aldrich	tagtatgttctccctgggcc
IER3-AS1_Probe8	Sigma Aldrich	gaggtagagggttgggggt
LacZ_Probe1	Sigma Aldrich	TCACGACGTTGTAAAACGAC
LacZ_Probe2	Sigma Aldrich	ATTAAGTTGGGTAACGCCAG
LacZ_Probe3	Sigma Aldrich	AGGTTACGTTGGTGTAGATG

LacZ_Probe4	Sigma Aldrich	AATGTGAGCGAGTAACAACC
LacZ_Probe5	Sigma Aldrich	GTAGCCAGCTTTCATCAACA
LacZ_Probe6	Sigma Aldrich	AATAATTCGCGTCTGGCCTT
LacZ_Probe7	Sigma Aldrich	AGATGAAACGCCGAGTTAAC
LacZ_Probe8	Sigma Aldrich	AATTCAGACGGCAAACGACT
**siRNA from sigma aldrich**		
**Name**	**Company**	**Target Sequence**
IER3-AS1 siRNA1	Sigma Aldrich	GTGATTGCCACATCTCGGA
IER3-AS1 siRNA2	Sigma Aldrich	GATGCTGGGTTCTGTGACT
IER3-AS1 siRNA3	Sigma Aldrich	GGGTTCTGTGACTCCAGGA
IER3 siRNA1	Sigma Aldrich	CTGGTGTTTCTTTGTGGTT
IER3 siRNA2	Sigma Aldrich	CGCTGTAGTGTTCTGAGTT
IER3 siRNA3	Sigma Aldrich	GTGCTGAGGTCCAGAGCGT
CCL20 siRNA1	Sigma Aldrich	SASI_Hs01_00124576
CCL20 siRNA2	Sigma Aldrich	SASI_Hs01_00124577
CCL20 siRNA3	Sigma Aldrich	SASI_Hs01_00124578
CXCL1 siRNA1	Sigma Aldrich	SASI_Hs01_00095009
CXCL1 siRNA2	Sigma Aldrich	SASI_Hs01_00095011
CXCL1 siRNA3	Sigma Aldrich	SASI_Hs02_00332014
hnRNPK siRNA1	Sigma Aldrich	SASI_Hs02_00358665
hnRNPK siRNA2	Sigma Aldrich	SASI_Hs02_00358664
hnRNPK siRNA3	Sigma Aldrich	SASI_Hs02_00358662
hnRNPC siRNA1	Sigma Aldrich	SASI_Hs02_00337755
hnRNPC siRNA2	Sigma Aldrich	SASI_Hs01_00010782
hnRNPC siRNA3	Sigma Aldrich	SASI_Hs02_00337754
SFN siRNA1	Sigma Aldrich	SASI_Hs01_00189928
SFN siRNA2	Sigma Aldrich	SASI_Hs01_00189929
Mission siRNA negative control	Sigma Aldrich	S1C001

Supplementary Table 4 – List of Plasmids, Drugs, reagents and software and algorithms used.

**Plasmids from addgene**		
**Name**	**Company**	**Identifier**
T7-RELA	Addgene	#21984
T7-RELA(S276A)	Addgene	#24154
PLKO.TRC (cloning vector for shRNA)	Addgene	#10878
LentisgRNA(MS2)Zeo (Cloning vector for sgRNA)	Addgene	#61427
Lenti dCAS9-VP64-Blast	Addgene	#61425
lentiMPH v2	Addgene	#89308
**Drugs and Reagents**		
Antibodies	**Company**	**Identifier**
Nutlin-3a	Selleckchem	S8059
FGF-2	Sigma Aldrich	SRP4037
ActinomycinD	Sigma Aldrich	A1410
Methylene Blue	Sigma Aldrich	M9140-25G
Corning Matrigel	Thermofischer Scientific	11573620
RIPA lysis Buffer	Sigma Aldrich	R0278
Protease & Phosphatases inhibitor cocktail	Thermofischer Scientific	78446
Skim Milk powder	Sigma Aldrich	70166
SuperSignal™ West Pico PLUS Chemiluminescent Substrate	Thermofischer Scientific	34580
Lipofectamine RNAi max	Thermofischer Scientific	10601435
Lipofectamine 2000	Thermofischer Scientific	12313563
**Software and algorithms**		
**Softwares**	**Company**	**Identifier**
GraphPad Prism	GraphPad	https://www.graphpad.com/scientific-software/prism/
ImageJ	NIH	https://imagej.nih.gov/ij/

Chemotaxis and Migration tool	Ibidi	https://ibidi.com/chemotaxis-analysis/171-chemotaxis-and-migration-tool.html?gclid=Cj0KCQiA-qGNBhD3ARIsAO_o7yndTIrYiAYt_Zf6zSAEzHiwNrE-g1XQqNRJa-ly0-ScW1ZVQaoloIMaAi1fEALw_wcB
Inkscape	N/A	https://inkscape.org/release/0.92.3/windows/
Zen	Zeiss	https://www.zeiss.com/microscopy/int/products/microscope-software/zen.html
IMARIS Imaging software	Oxford instruments	https://imaris.oxinst.com/products/imaris-for-cell-biologists?gclid=Cj0KCQiA-qGNBhD3ARIsAO_o7ykO6PENdQyqCHZLUsQkyen95EerGxIgojU-sDutTgvZzha_sqTTuhsaAmV8EALw_wcB

Supplementary Table 5 – List of Antibodies and dilutions used for western blot, ChRIP, dot blot, Immunostaining and IHC experiments.

Western blot antibodies			
**Antibodies**	**Company**	**Catalog No.**	**Dilution**
pAKT (Ser473)	cell signaling	4060S	1:2000
AKT	cell signaling	4691S	1:1000
pERK	cell signaling	9101S	1:1000
ERK	cell signaling	4695S	1:1000
IER3	Abcam	ab65152	1:1000
GAPDH	Santa Cruz Biotech	sc-25778	1:1000
ZFP36	Proteintech	12737-1-AP	1:1000
PARP	cell signaling	9532S	1:1000
Caspase3	Abcam	ab32042	1:500
CLL20	Novus Biologicals	MAB360	1:1000
CXCL1	Novus Biologicals	AF275	1:1000
p65	cell signaling	8242S	1:1000

p(Ser276) p65	Millipore	AB3375	1:1000
hnRNPK	cell signaling	9081S	1:1000
RIG1	Thermofischer Scientific	PA5-23497	1:500
MDA5	Thermofischer Scientific	700360	1:500
H3	Abcam	ab176842	1:1000
**ChRIP antibodies**			
**Antibodies**	**Company**	**Identifier**	
IgG	Millipore	12-370	1:500
hnRNPK	Thermofisher	PA5-27522	1:500
Anti-dsRNA J2	Jena Bioscience	RNT-SCI-10010200	1:500
**Dot blot and Immunostaining antibodies**			
**Antibodies**	**Company**	**Identifier**	
DAP1	cell signaling	2282S	1:500
RPS3	Novus	NBP1-33691	1:250
Anti-dsRNA J2	Jena Bioscience	RNT-SCI-10010200	1:250
**IHC antibodies**			
Ki67	Abcam	Ab16667	1:500

Supplementary Table 6 – Validation for all the antibodies used in this study

**Western blot antibodies**				
**Antibodies**	**Company**	**Identifier**	**Validation**	
			**Company**	**References**
pAKT (Ser473)	cell signaling	4060S	https://www.cellsignal.com/product/productDetail.jsp?productId=4060	Nikos Koundouros, et. al., Cell., 2020
AKT	cell signaling	4691S	https://www.cellsignal.com/products/primary-antibodies/akt-pan-c67e7-rabbit-mab/4691	Johanna Wagner, et. al., Cell., 2019
pERK	cell signaling	9101S	https://www.cellsignal.com/product/productDetail.jsp?productId=9101	Suji Han, et. al.., Cancers., 2019
ERK	cell signaling	4695S	https://www.cellsignal.com/product/productDetail.jsp?productId=4695	Zaigham M Khan, et. al., Nature., 2020
IER3	Abcam	ab65152	https://www.abcam.com/iex1ier3-antibody-ab65152.html	Zhou Q et al., Int J Mol Sci., 2017
GAPDH	Santa Cruz Biotech	sc-25778	https://www.scbt.com/sv/p/gapdh-antibody-fl-335	Llorian, M. et al., Nucleic Acids Res., 2016.
ZFP36	Proteintech	12737-1-AP	https://www.ptglab.com/products/ZFP36-Antibody-12737-1-AP.htm	Yuan Wang et al., Sci Rep., 2017

PARP	cell signaling	9532S	https://www.cellsignal.com/product/productDetail.jsp?productId=9532	Maximilien Tailler, et. al., Cell Death Differ., 2019
Caspase3	Abcam	ab32042	https://www.abcam.com/cleaved-caspase-3-antibody-e83-77-ab32042.html	Kumar A et al., Cell Rep., 2020
CLL20	Novus Biologicals	MAB360	https://www.novusbio.com/products/ccl20-mip-3-alpha-antibody-67310_mab360	B Soto et al., Sci Rep., 2017
CXCL1	Novus Biologicals	AF275	https://www.novusbio.com/products/cxcl1-gro-alpha-kc-cinc-1-antibody_af275	Keane MP et al., J. Immonol., 2002
p65	cell signaling	8242S	https://www.cellsignal.com/product/productDetail.jsp?productId=8242	Hebah A Sindi, et. al., Nat Commun., 2020
p(Ser276) p65	Millipore	AB3375	https://www.merckmillipore.com/SE/en/product/Anti-NFB-p65-Antibody-phospho-specific-Ser276,MM_NF-AB3375?	Ashikawa, Kazuhiro, et al., J. Immunol., 2002
hnRNPK	cell signaling	9081S	https://www.cellsignal.com/product/productDetail.jsp?productId=9081	Helen Wong, et. al., Elife., 2020
RIG1	Thermofischer Scientific	PA5-23497	https://www.thermofisher.com/antibody/product/RIG-I-Antibody-Polyclonal/PA5-23497	Cell Treatment Antibody Validation. https://www.thermofisher.com/antibody/product/RIG-I-Antibody-Polyclonal/PA5-23497
MDA5	Thermofischer Scientific	700360	https://www.thermofisher.com/antibody/product/MDA5-Antibody-clone-33H12L34-Recombinant-Monoclonal/700360	Shao W et al., JCI insight., 2018
H3	Abcam	ab176842	https://www.abcam.com/histone-h3-antibody-epr16987-nuclear-marker-and-chip-grade-ab176842.html	Bhattacharyya T et al., Curr Biol., 2019
**ChRIP antibodies**				
**Antibodies**	**Company**	**Identifier**	**Validation**	
			**Company**	**References**
IgG	Millipore	12-370	https://www.merckmillipore.com/SE/en/product/Normal-Rabbit-IgG,MM_NF-12-370	Frank, CL et al., Nat Neurosc., 2015
hnRNPK	Thermofisher	PA5-27522	https://www.thermofisher.com/antibody/product/hnRNP-K-Antibody-Polyclonal/PA5-27522	This Antibody was verified by Knockdown to ensure that the antibody binds to the antigen stated.
Anti-dsRNA J2	Jena Bioscience	RNT-SCI-10010200	https://www.jenabioscience.com/rna-technologies/rna-analysis-detection/dsrna-detection/rnt-sci-10010-anti-dsrna-monoclonal-antibody-j2	Schönborn et al., Nucleic Acids Res., 1991
**Dot blot and Immunostaining antibodies**				

Antibodies	Company	Identifier	Validation	
			Company	References
DAP1	cell signaling	2282S	https://www.cellsignal.com/product/productDetail.jsp?productId=2282	Rafal Sadej, et. al., J Cell Sci., 2018
RPS3	Novus	NBP1-33691	https://www.novusbio.com/products/rps3-antibody_nbp1-33691	This Antibody was verified by Knockdown to ensure that the antibody binds to the antigen stated.
Anti-dsRNA J2	Jena Bioscience	RNT-SCI-10010200	https://www.jenabioscience.com/rna-technologies/rna-analysis-detection/dsrna-detection/rnt-sci-10010-anti-dsrna-monoclonal-antibody-j2	Schönborn et al., Nucleic Acids Res., 1991
Ki67	Abcam	Ab16667	https://www.abcam.com/ki67-antibody-sp6-ab16667.html	Messal HA et al., Nat Protoc., 2021
