# Supplementary material for: HnRNPK maintains single strand RNA through controlling double-strand RNA in mammalian cells
Source: Nat Commun. 2022 Aug 29;13:4865. doi: 10.1038/s41467-022-32537-0 (PMC9424213; doi:10.1038/s41467-022-32537-0)
Supplement: Supplementary file 3 — Description of Additional Supplementary Files [file 41467_2022_32537_MOESM3_ESM.pdf]

Descriptions of Additional Supplementary Files

Supplementary data file 1 – List of all the significant Differentially expressed genes (DEGs) for FGF-2 induction and GO enrichment analysis pathways for FGF-2 upregulated, down-regulated and all genes from FGF-2 induction for BT-549, HeLa and HEK293 cell lines. 2.

Supplementary data file 2 - List of all the significant DEGs and Biological pathways for IER3sh and IER3-AS1sh KD respectively for all three cell lines; HEK293, HeLa and BT-549. 3.

Supplementary data file 3 - List of all the significant DEGs and GO enrichment analysis pathways for upregulated, down-regulated and all genes from siHNRNPK_Total RNA, siHNRPK_cytoplasmicRNA and siHNRPK_nuclearRNA transcriptomic data in HeLa cell line.
