# Supplementary material for: HnRNPK maintains single strand RNA through controlling double-strand RNA in mammalian cells
Source: Nat Commun. 2022 Aug 29;13:4865. doi: 10.1038/s41467-022-32537-0 (PMC9424213; doi:10.1038/s41467-022-32537-0)
Supplement: Supplementary file 7 — Reporting Summary [file 41467_2022_32537_MOESM7_ESM.pdf]

nature portfolio

Corresponding author(s): Meena Kanduri and Chandrasekhar Kanduri

Last updated by author(s): Jun 30, 2022

Reporting Summary

Nature Portfolio wishes to improve the reproducibility of the work that we publish. This form provides structure for consistency and transparency in reporting. For further information on Nature Portfolio policies, see our Editorial Policies and the Editorial Policy Checklist.

Statistics

For all statistical analyses, confirm that the following items are present in the figure legend, table legend, main text, or Methods section.

n/a	Confirmed	
☐	☒	The exact sample size (*n*) for each experimental group/condition, given as a discrete number and unit of measurement
☐	☒	A statement on whether measurements were taken from distinct samples or whether the same sample was measured repeatedly
☐	☒	The statistical test(s) used AND whether they are one- or two-sided<br>*Only common tests should be described solely by name; describe more complex techniques in the Methods section.*
☒	☐	A description of all covariates tested
☒	☐	A description of any assumptions or corrections, such as tests of normality and adjustment for multiple comparisons
☐	☒	A full description of the statistical parameters including central tendency (e.g. means) or other basic estimates (e.g. regression coefficient) AND variation (e.g. standard deviation) or associated estimates of uncertainty (e.g. confidence intervals)
☐	☒	For null hypothesis testing, the test statistic (e.g. *F*, *t*, *r*) with confidence intervals, effect sizes, degrees of freedom and *P* value noted<br>*Give P values as exact values whenever suitable.*
☒	☐	For Bayesian analysis, information on the choice of priors and Markov chain Monte Carlo settings
☒	☐	For hierarchical and complex designs, identification of the appropriate level for tests and full reporting of outcomes
☒	☐	Estimates of effect sizes (e.g. Cohen's *d*, Pearson's *r*), indicating how they were calculated

Our web collection on statistics for biologists contains articles on many of the points above.

Software and code

Policy information about availability of computer code

Data collection	For RNA sequencing, two independent shRNAs transduced cells were sequenced in duplicate sets using Illumina Sequencing Platform. Directional mRNA library preparation (poly A enrichment) was performed using NovaSeq PE150 (12G raw data per sample).
Data analysis	The Illumina sequenced reads were aligned to hg38 (GRCh38) version of the genome using HISAT 2.2.1 aligner and quantified using feature Counts (Subread-1.4.5). The differential expression of treated and untreated samples was performed using DESeq using R package. Adjusted p-value of 0.05 by Benjamini-Hochberg method and log2FC of 1 is considered as significant. Functional enriched biological pathways of differentially expressed protein coding genes were determined using GeneSCF (FDR <=0.05). The differentially expressed genes were considered significant with adjusted p-value of 0.05 by Benjamini-Hochberg. The image analysis was performed using Imaris microscopy image analysis software 9.8.2 version. Statistical analyses were performed using GraphPad Prism 8.4 version.

For manuscripts utilizing custom algorithms or software that are central to the research but not yet described in published literature, software must be made available to editors and reviewers. We strongly encourage code deposition in a community repository (e.g. GitHub). See the Nature Portfolio guidelines for submitting code & software for further information.

Data

Policy information about availability of data

All manuscripts must include a data availability statement. This statement should provide the following information, where applicable:

- Accession codes, unique identifiers, or web links for publicly available datasets
- A description of any restrictions on data availability
- For clinical datasets or third party data, please ensure that the statement adheres to our policy

The RNA sequencing which is listed in Supplementary file 1-3 are deposited in Gene expression omnibus data base and the GEO accession number for data generated for this paper is GSE190212. The source data for all the figures will be submitted prior to the publication.

Human research participants

Policy information about studies involving human research participants and Sex and Gender in Research.

Reporting on sex and gender	*Use the terms sex (biological attribute) and gender (shaped by social and cultural circumstances) carefully in order to avoid confusing both terms. Indicate if findings apply to only one sex or gender; describe whether sex and gender were considered in study design whether sex and/or gender was determined based on self-reporting or assigned and methods used. Provide in the source data disaggregated sex and gender data where this information has been collected, and consent has been obtained for sharing of individual-level data; provide overall numbers in this Reporting Summary. Please state if this information has not been collected. Report sex- and gender-based analyses where performed, justify reasons for lack of sex- and gender-based analysis.*
Population characteristics	*Describe the covariate-relevant population characteristics of the human research participants (e.g. age, genotypic information, past and current diagnosis and treatment categories). If you filled out the behavioural & social sciences study design questions and have nothing to add here, write "See above."*
Recruitment	*Describe how participants were recruited. Outline any potential self-selection bias or other biases that may be present and how these are likely to impact results.*
Ethics oversight	*Identify the organization(s) that approved the study protocol.*

Note that full information on the approval of the study protocol must also be provided in the manuscript.

Field-specific reporting

Please select the one below that is the best fit for your research. If you are not sure, read the appropriate sections before making your selection.

☒ Life sciences ☐ Behavioural & social sciences ☐ Ecological, evolutionary & environmental sciences

For a reference copy of the document with all sections, see nature.com/documents/nr-reporting-summary-flat.pdf

Life sciences study design

All studies must disclose on these points even when the disclosure is negative.

Sample size	Sample sizes for cell based assays were chosen according to the standards in the field or by previously published studies that have similar methods (at least two to three independent biological replicates for each condition) which gave sufficient statistical power for the effect sizes of interest. They were not predetermined based upon statistical methods given the inability to predict effect size for the cell experiments employed herein. The exact sample sizes are given in the legends of each figures in the manuscript. Minimum sample size for animal studies were according to the ethical permit (No: 5.8.18-02708/2017), reviewed and approved by the Animal Ethical Review Board, University of Gothenburg, Sweden. The sample citations includes:<br>(1) Mondal, T. et al. MEG3 long noncoding RNA regulates the TGF-beta pathway genes through formation of RNA-DNA triplex structures. Nature communications 6, 7743 (2015).<br>(2) Li, J. et al. HNRNPK maintains epidermal progenitor function through transcription of proliferation genes and degrading differentiation promoting mRNAs. Nature communications 10, 4198 (2019).<br>(3) Pandey, R.R. et al. Kcnq1ot1 antisense noncoding RNA mediates lineage-specific transcriptional silencing through chromatin-level regulation. Molecular cell 32, 232-246 (2008).
Data exclusions	No data were excluded from the analysis
Replication	All the gene expression analysis, RIPs, ChOPs etc and functional assays such as cell proliferation assays, apoptosis and cell cycle analysis were performed using 2 to 3 biological replicates and the data in all replicates were consistently reproducible. All the immunoblots were replicated in independent experiments and the replicate immunoblots showed the similar results. RNA sequencing for knockdown samples and FGF-2 treated samples were performed in two biological replicates. Sample correlation cluster analysis was used to assess successful reproducibility. All experiments were consistently reproducible. All graphs include individual data points from biological replicates.

Randomization	The mice were randomly allocated into the cages before the treatment. The 4-6 weeks old male and female mice were allocated randomly into two groups (control sh group and IER3-AS1 sh group). All experiments were performed with appropriate negative and positive controls in keeping with the standards of the field.
Blinding	Not applicable.

Reporting for specific materials, systems and methods

We require information from authors about some types of materials, experimental systems and methods used in many studies. Here, indicate whether each material, system or method listed is relevant to your study. If you are not sure if a list item applies to your research, read the appropriate section before selecting a response.

Materials & experimental systems

n/a	Involved in the study	
☐	☒	Antibodies
☐	☒	Eukaryotic cell lines
☒	☐	Palaeontology and archaeology
☐	☒	Animals and other organisms
☒	☐	Clinical data
☒	☐	Dual use research of concern

Methods

n/a	Involved in the study	
☒	☐	ChIP-seq
☒	☐	Flow cytometry
☒	☐	MRI-based neuroimaging

Antibodies

Antibodies used

Antibodies used for Western blot: pAKT (Ser473), cell signaling, 4060S (1:2000 dilution); AKT, cell signaling, 4691S (1:1000 dilution); pERK, cell signaling, 9101S (1:1000 dilution); ERK, cell signaling, 4695S (1:1000 dilution); IER3, Abcam, ab65152 (3ug/ml); GAPDH, Santa Cruz Biotech, sc-25778(1:1000 dilution); ZFP36, Proteintech, 12737-1-AP (1:1000 dilution); PARP, cell signaling, 9532S (1:1000 dilution); Caspase3, Abcam, ab32042 (1:500 dilution); CLL20, Novus Biologicals, MAB360 (lug/ml); CXCLl, Novus Biologicals, AF275 (0.lug/ml); p65, cell signaling, 8242S (1:1000 dilution); phospho Ser276 (l:1000Ser276)p65, Millipore, AB3375 (1:1000 dilution); hnRNPK, cell signaling, 9081S (1:1000 dilution); RIGl, Thermofischer Scientific, PAS-23497 (3ug/ml); MDA5 (700360), Thermofischer Scientific, (Sug/ml); H3, Abcam, ab176842 (lug/ml).
Antibodies used for RIP assay: lgG, Millipore, 12-370; hnRNPK, Thermofisher Scientific, PAS-27522; Anti-dsRNA J2, Jena Bioscience, RNT-SCl-10010200.
Dot blot and lmmunostaining antibodies: RPS3, Novus, NBPl-33691; Anti-dsRNA J2, Jena Bioscience, RNT-SCl-10010200; Ki67; Abcam, Ab16667.
Nucleocounter staining antibodies: Annexin V, Thermofischer Scientific, A13201; Pl, Chemometec, 910-3016.

Validation

We selected these antibodies considering their wide use by the research community. In addition, they have been validated by the manufacturer. The detail information of each antibody with references is given below:
pAKT (Ser473) (cellsignaling): https://www.cellsignal.com/product/productDetail.jsp?productId=4060, Nikos Koundouros, et. al., Cell., 2020.
AKT (cell signaling): https://www.cellsignal.com/products/primary-antibodies/akt-pan-c67e7-rabbit-mab/4691; Johanna Wagner, et. al., Cell., 2019.
pERK(cell signaling): https://www.cellsignal.com/product/productDetail.jsp?productId=9101,Suji Han, et. al.., Cancers., 2019.
ERK (cell signaling): https://www.cellsignal.com/product/productDetail.jsp?productId=4695, Zaigham M Khan, et. al., Nature., 2020.
IER3(Abcam): https://www.abcam.com/iex1ier3-antibody-ab65152.html, Zhou Q et al., Int J Mol Sci., 2017.
GAPDH(Santa Cruz Biotech): https://www.scbt.com/sv/p/gapdh-antibody-fl-335,Llorian, M. et al., Nucleic Acids Res., 2016.
ZFP36(Proteintech): https://www.ptglab.com/products/ZFP36-Antibody-12737-1-AP.htm, Yuan Wang et al., Sci Rep., 2017.
PARP(cell signaling): https://www.cellsignal.com/product/productDetail.jsp?productId=9532, Maximilien Tailler, et. al., Cell Death Differ., 2019.
Caspase3(Abcam):https://www.abcam.com/cleaved-caspase-3-antibody-e83-77-ab32042.html, Kumar A et al., Cell Rep., 2020.
CLL20(NovusBiologicals): https://www.novusbio.com/products/ccl20-mip-3-alpha-antibody-67310_mab360,B Soto et al., Sci Rep., 2017.
CXCL1(Novus Biologicals): https://www.novusbio.com/products/cxcl1-gro-alpha-kc-cinc-1-antibody_af275,Keane MP et al., J. Immonol., 2002.
p65(cell signaling): https://www.cellsignal.com/product/productDetail.jsp?productId=8242, Hebah A Sindi, et. al., Nat Commun., 2020.
p(Ser276) p65 (Millipore): https://www.merckmillipore.com/SE/en/product/Anti-NFB-p65-Antibody-phospho-specific-Ser276,MM_NF-AB3375?, Ashikawa, Kazuhiro, et al., J. Immunol., 2002.
hnRNPK(cell signaling): https://www.cellsignal.com/product/productDetail.jsp?productId=9081, Helen Wong, et. al., Elife., 2020.
RIG1(Thermofischer Scientific): https://www.thermofisher.com/antibody/product/RIG-I-Antibody-Polyclonal/PA5-23497, Cell Treatment Antibody Validation.
MDA5(Thermofischer Scientific): https://www.thermofisher.com/antibody/product/MDA5-Antibody-clone-33H12L34-Recombinant-Monoclonal/700360, Shao W et al., JCI insight., 2018.
H3(Abcam): https://www.abcam.com/histone-h3-antibody-epr16987-nuclear-marker-and-chip-grade-ab176842.html, Bhattacharyya T et al., Curr Biol., 2019.

IgG (Millipore): https://www.merckmillipore.com/SE/en/product/Normal-Rabbit-IgG,MM_NF-12-370, Frank, CL et al., Nat Neurosc., 2015.
hnRNPK (Thermofisher): https://www.thermofisher.com/antibody/product/hnRNP-K-Antibody-Polyclonal/PA5-27522, This Antibody was verified by Knockdown to ensure that the antibody binds to the antigen stated.
Anti-dsRNA J2 (Jena Bioscience): https://www.jenabioscience.com/rna-technologies/rna-analysis-detection/dsrna-detection/rnt-sci-10010-anti-dsrna-monoclonal-antibody-j2, Schönborn et al., Nucleic Acids Res., 1991.
DAP1(cell signaling): https://www.cellsignal.com/product/productDetail.jsp?productId=2282, Rafal Sadej, et. al., J Cell Sci., 2018.
RPS3(Novus): https://www.novusbio.com/products/rps3-antibody_nbp1-33691, This Antibody was verified by Knockdown to ensure that the antibody binds to the antigen stated.
Ki67(Abcam): https://www.abcam.com/ki67-antibody-sp6-ab16667.html, Messal HA et al., Nat Protoc., 2021.

Eukaryotic cell lines

Policy information about cell lines and Sex and Gender in Research

Cell line source(s)	HeLa (300194), A549 (300114), BT-549 (300132), HEK293T (300189) cell lines were purchased from CLS Cell Lines Service. GmbH
Authentication	All the cell lines were authenticated by CLS Cell Lines Service when we purchased them. CLS uses STR analysis to authenticate their cell lines. We did not independently authenticate them.
Mycoplasma contamination	All cell lines were confirmed mycoplasma negative. We conduct routine mycoplasma testing in the laboratory every month.
Commonly misidentified lines (See ICLAC register)	None of the commonly misidentified cell lines were employed in this study.

Animals and other research organisms

Policy information about studies involving animals; ARRIVE guidelines recommended for reporting animal research, and Sex and Gender in Research

Laboratory animals	For mouse xenograft experiment we used total 10 mice of both sexes (male and female) were selected (5 each in each group). 4 to 6 week old NSG mice (Charles River, France) were subcutaneously injected on the dorsal back region for tumor formation. All the experimental animals were kept in animal house facility at the University of Gothenburg. All the animals were kept to standard day/night cycle, ambient temperature and humidity. The light was on between 07.00-19:00 and off between 19:00-07:00, the normal ambient temperature was 21 degrees Celsius and humidity was 45-70% Rh value.<br>NSG mice are kept in individually ventilated cages (IVC) as per the standard routines maintained by Experimental Biomedicine (EBM), University of Gothenburg, Sweden.
Wild animals	No wild animals are used in this study
Reporting on sex	Animal sex were not consider in this study. We used both sexes animals in this study.
Field-collected samples	No field collected samples were used.
Ethics oversight	We performed all animal experiments according to the ethical permit (No: 5.8.18-02708/2017), reviewed and approved by the Animal Ethical Review Board, University of Gothenburg, Sweden.

Note that full information on the approval of the study protocol must also be provided in the manuscript.
