# Supplementary material for: HnRNPK maintains single strand RNA through controlling double-strand RNA in mammalian cells
Source: Nat Commun. 2022 Aug 29;13:4865. doi: 10.1038/s41467-022-32537-0 (PMC9424213; doi:10.1038/s41467-022-32537-0)
Supplement: Supplementary file 8 — Source Data [file 41467_2022_32537_MOESM8_ESM.zip › Source data folder/Source file for WB of main figures_20220710.pdf]

Figure 1D

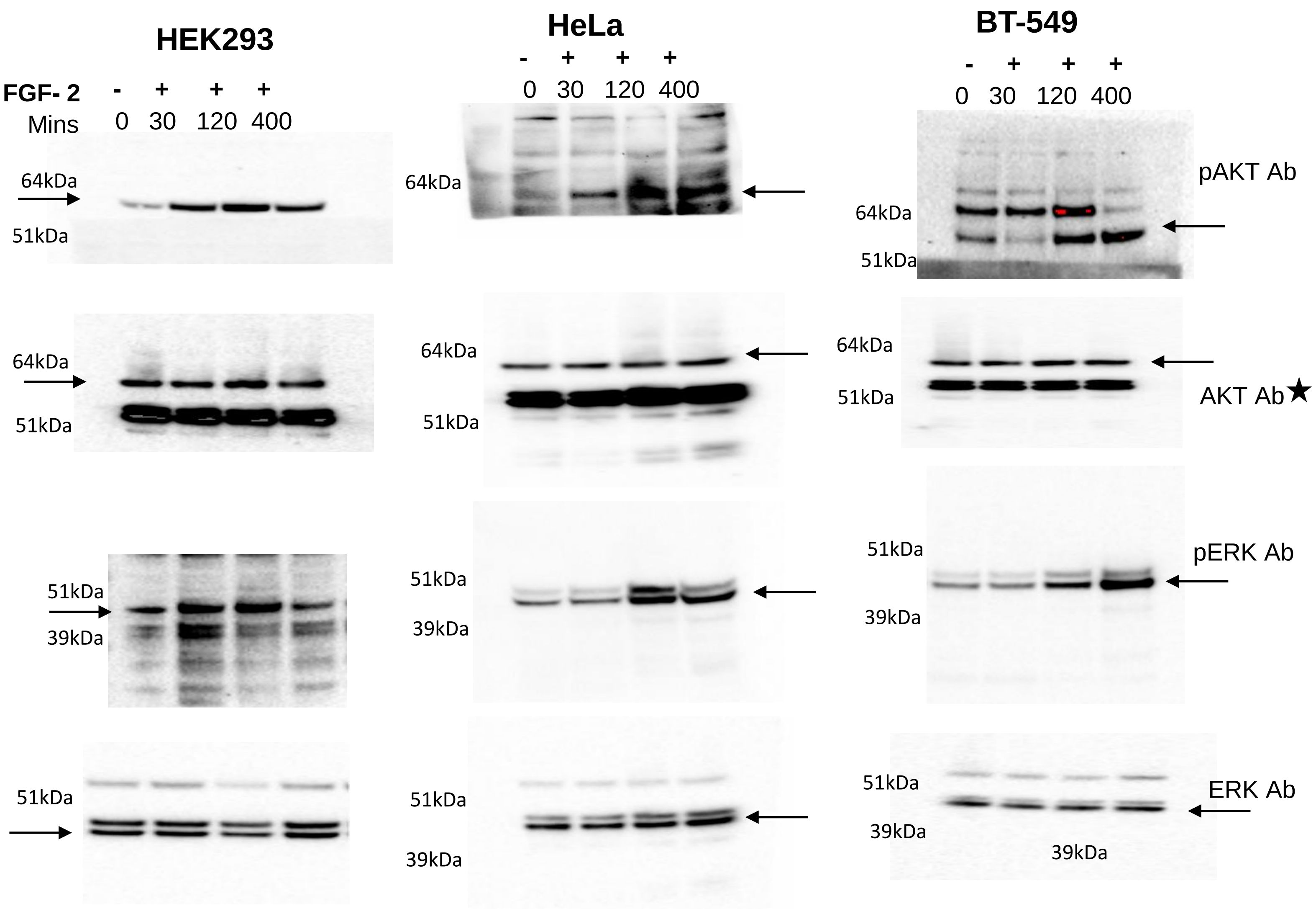

★ (Total ERK blots were stripped and probed with antibodies for total AKT levels).

Figure 1I

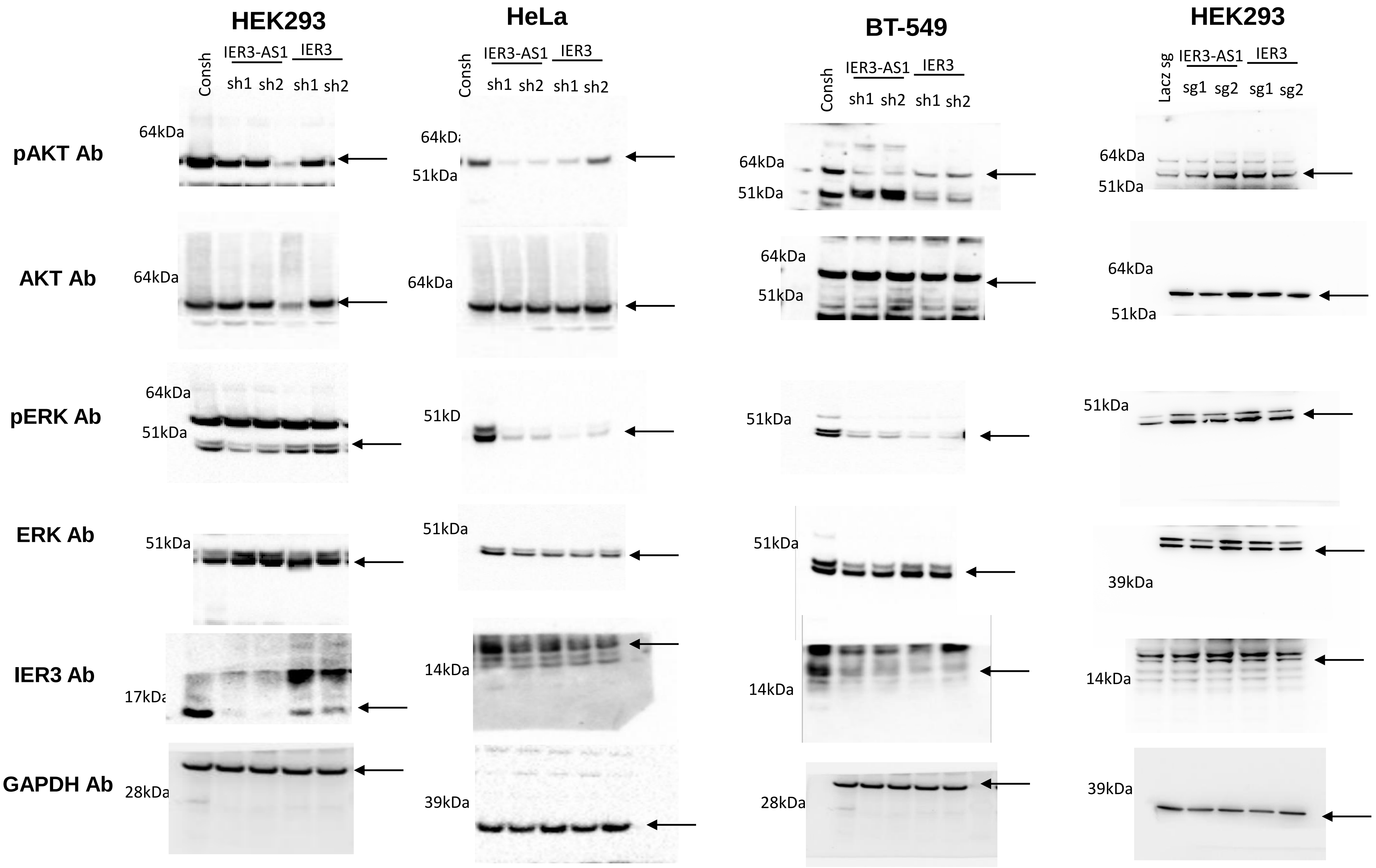
HEK293
HeLa
BT-549
HEK293
Consh
IER3-AS1
IER3
sh1
sh2
sh1
sh2
Lacz sg
sg1
sg2
sg1
sg2
pAKT Ab
AKT Ab
pERK Ab
ERK Ab
IER3 Ab
GAPDH Ab
64kDa
51kDa
39kDa
28kDa
17kDa
14kDa

Figure 2C

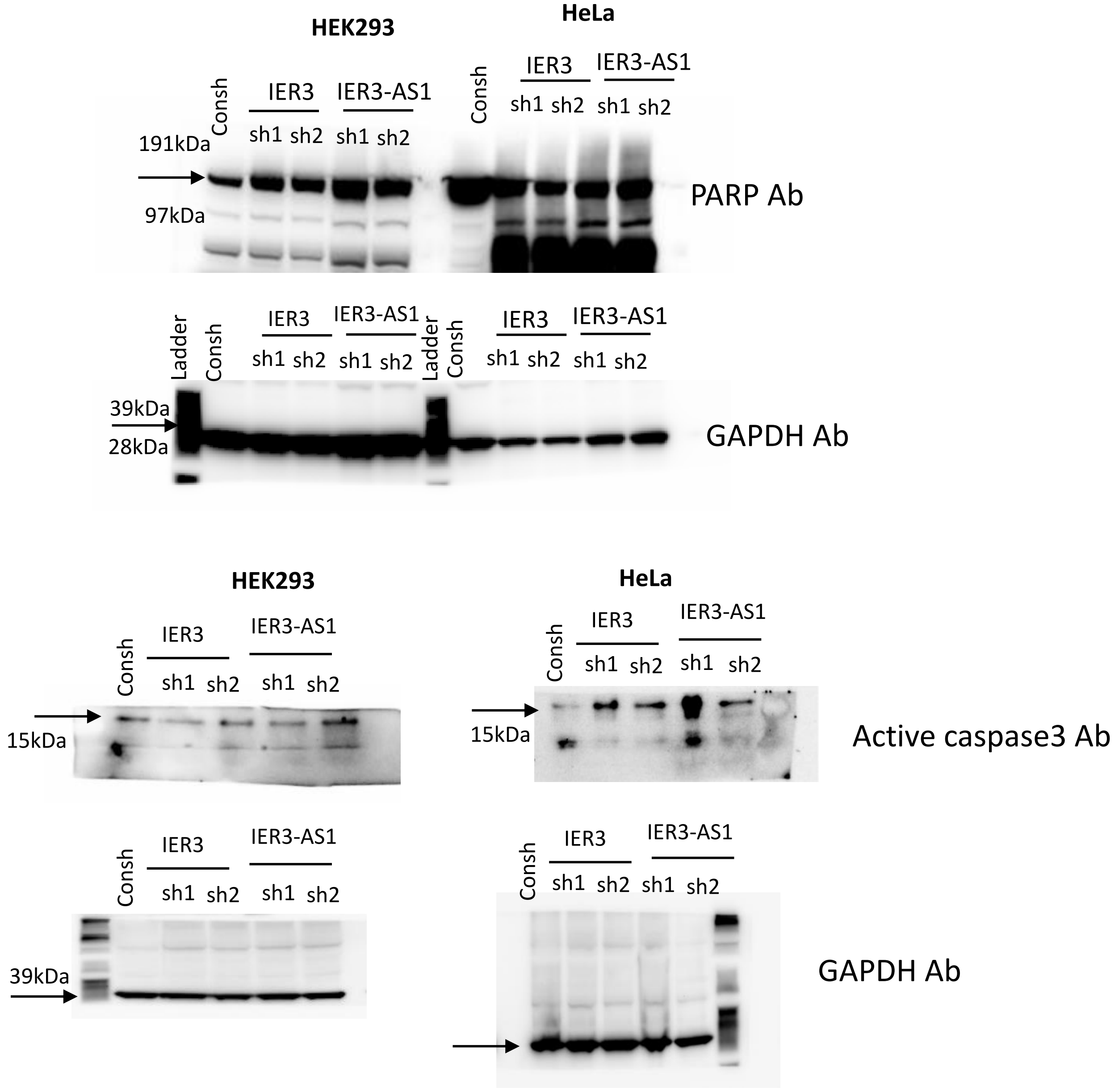
HEK293
HeLa
Consh
IER3
IER3-AS1
sh1 sh2 sh1 sh2
191kDa
97kDa
PARP Ab
Ladder
39kDa
28kDa
GAPDH Ab
HEK293
HeLa
15kDa
Active caspase3 Ab
39kDa
GAPDH Ab

Figure 4J

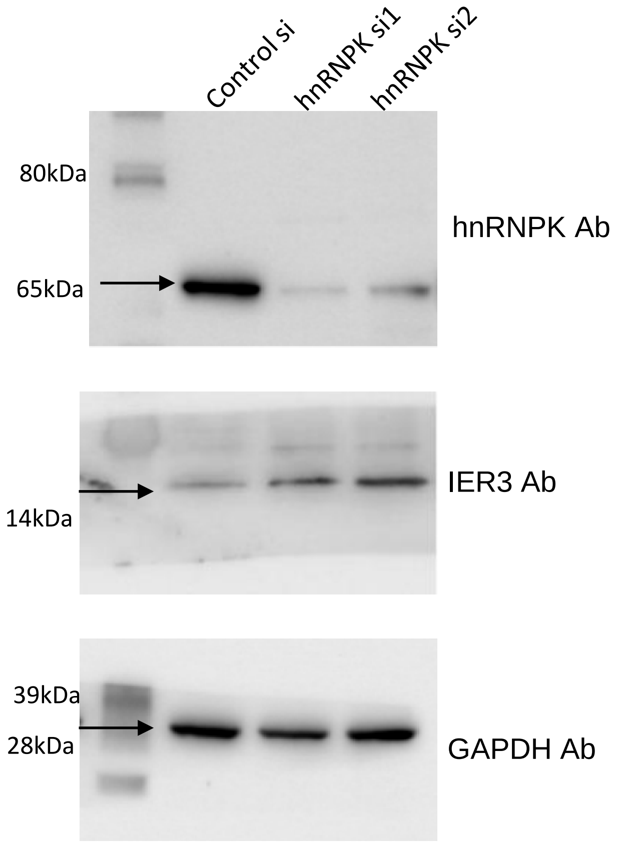
Control si
hnRNPK si1
hnRNPK si2
80kDa
65kDa
hnRNPK Ab
14kDa
IER3 Ab
39kDa
28kDa
GAPDH Ab

Figure 4K

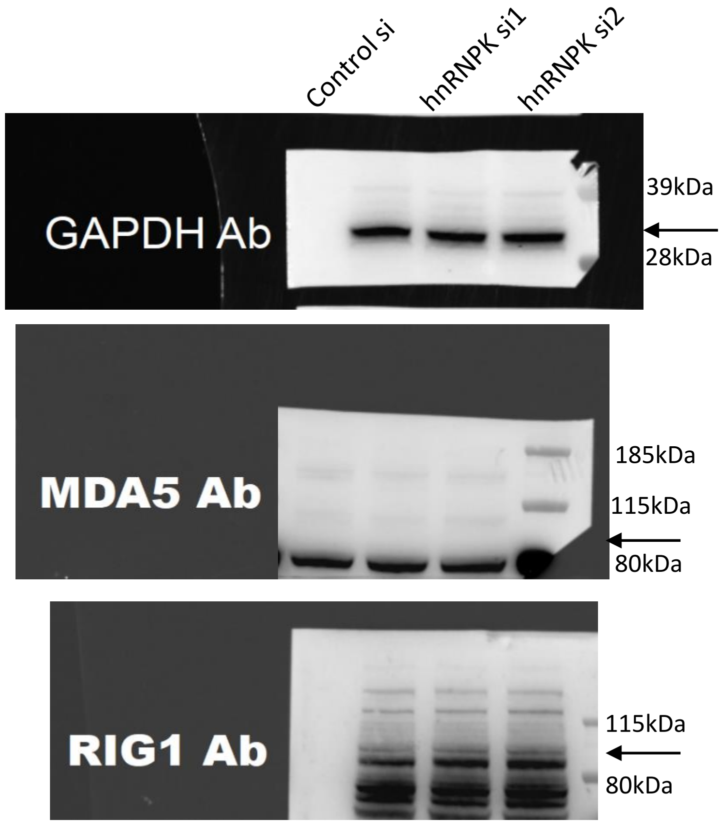

Control si
hnRNPK si1
hnRNPK si2
GAPDH Ab
39kDa
28kDa
MDA5 Ab
185kDa
115kDa
80kDa
RIG1 Ab
115kDa
80kDa

Figure 5A

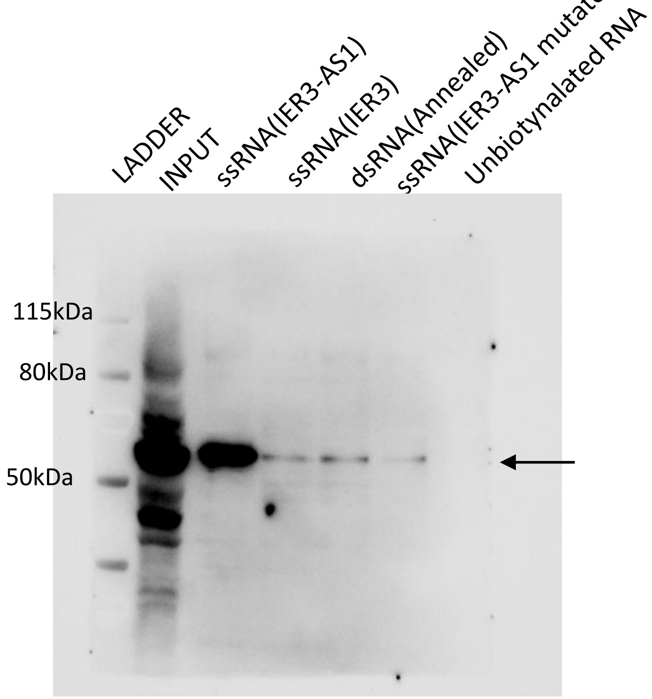

hnRNPK Ab

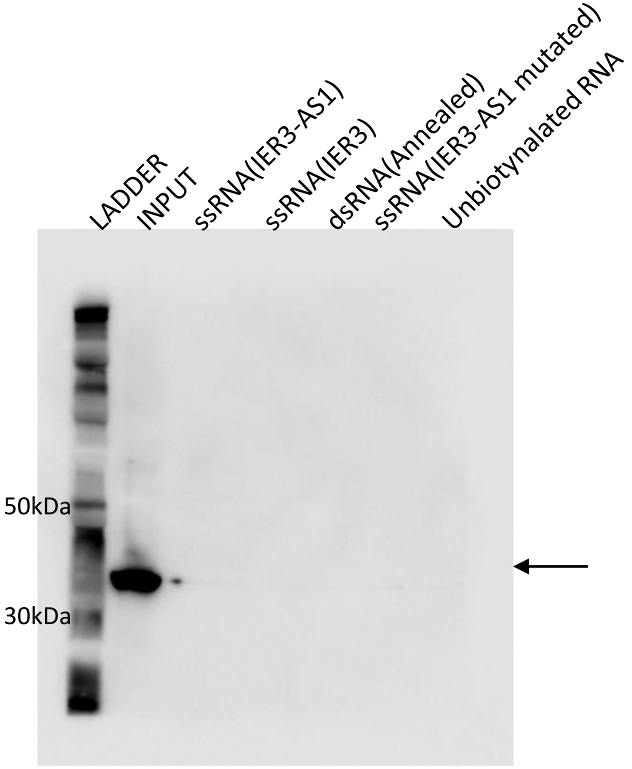

GAPDH Ab

Figure 6D

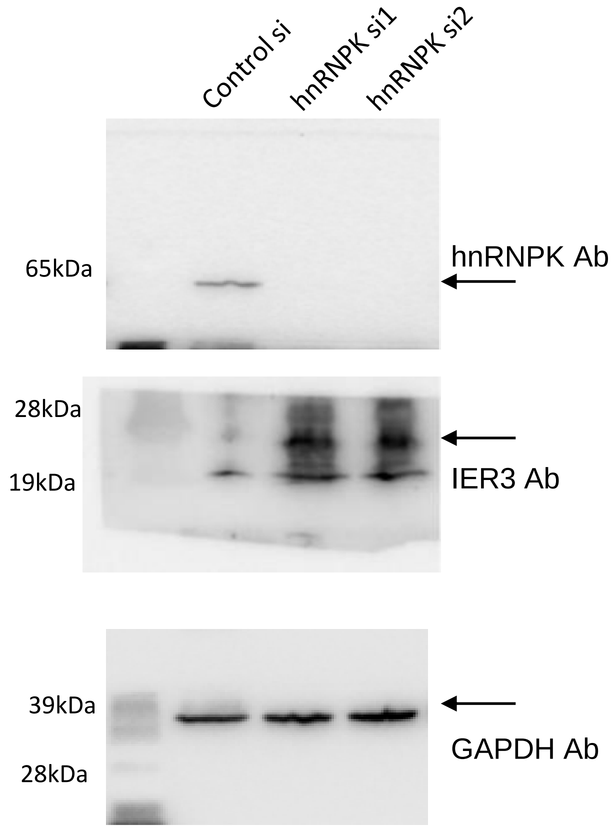
Control si
hnRNPK si1
hnRNPK si2
65kDa
hnRNPK Ab
28kDa
19kDa
IER3 Ab
39kDa
28kDa
GAPDH Ab

Figure 7B

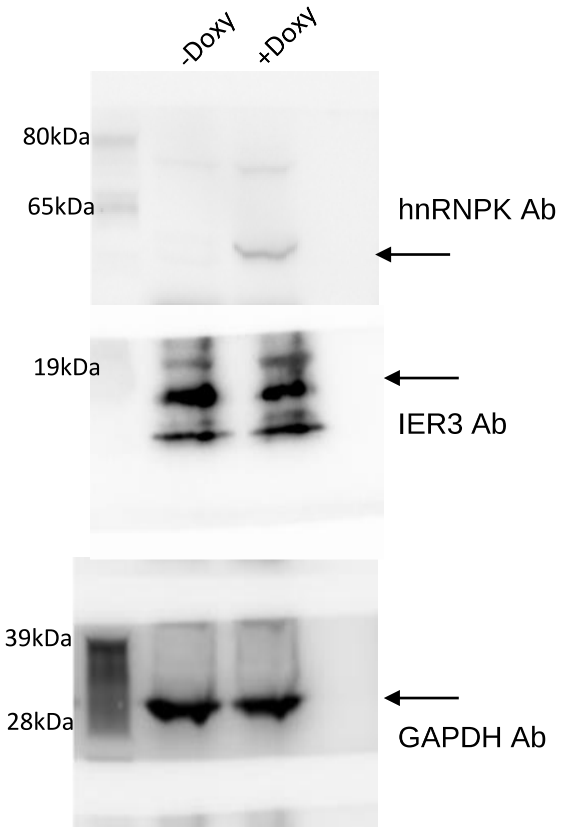
-Doxy
+Doxy
80kDa
65kDa
hnRNPK Ab
19kDa
IER3 Ab
39kDa
28kDa
GAPDH Ab
