# Supplementary material for: HnRNPK maintains single strand RNA through controlling double-strand RNA in mammalian cells
Source: Nat Commun. 2022 Aug 29;13:4865. doi: 10.1038/s41467-022-32537-0 (PMC9424213; doi:10.1038/s41467-022-32537-0)

Supplementary source data file for western blots

Supplementary Figure 4G

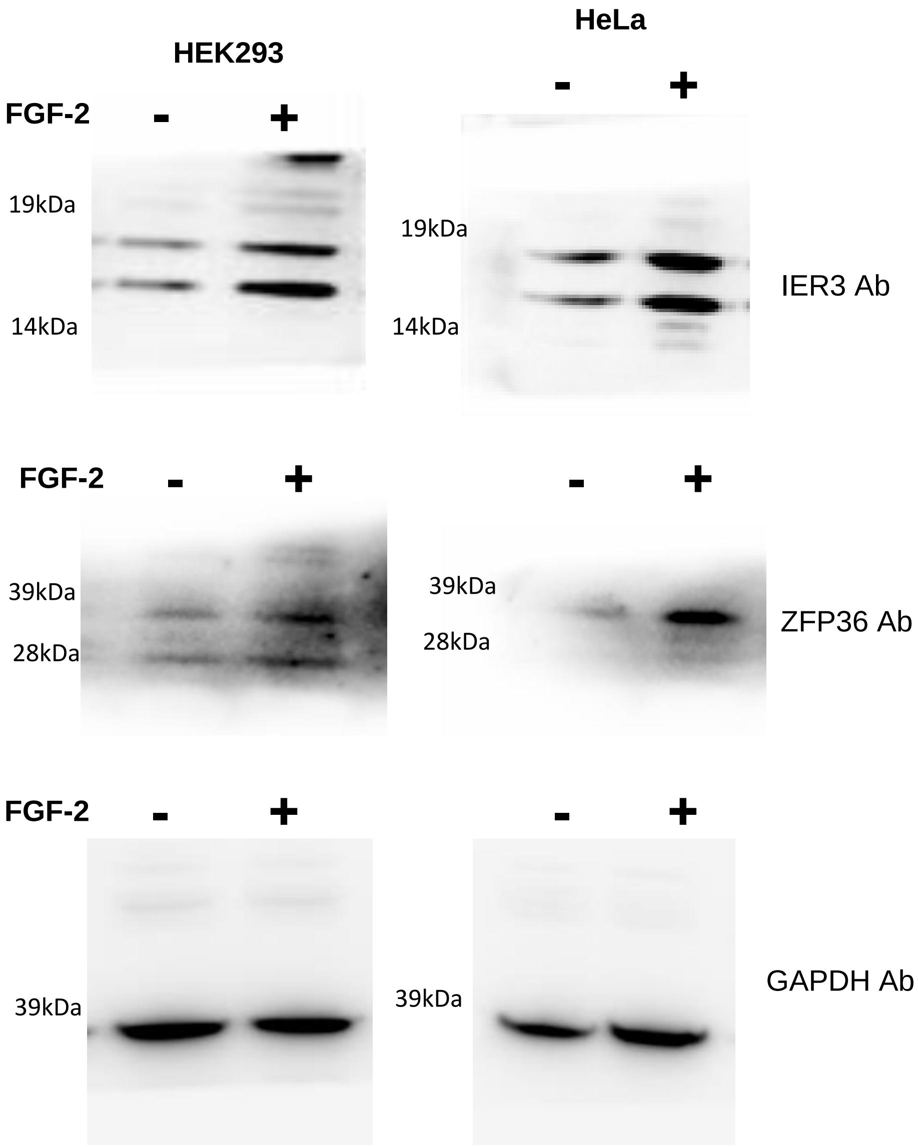

Supplementary Figure 4H

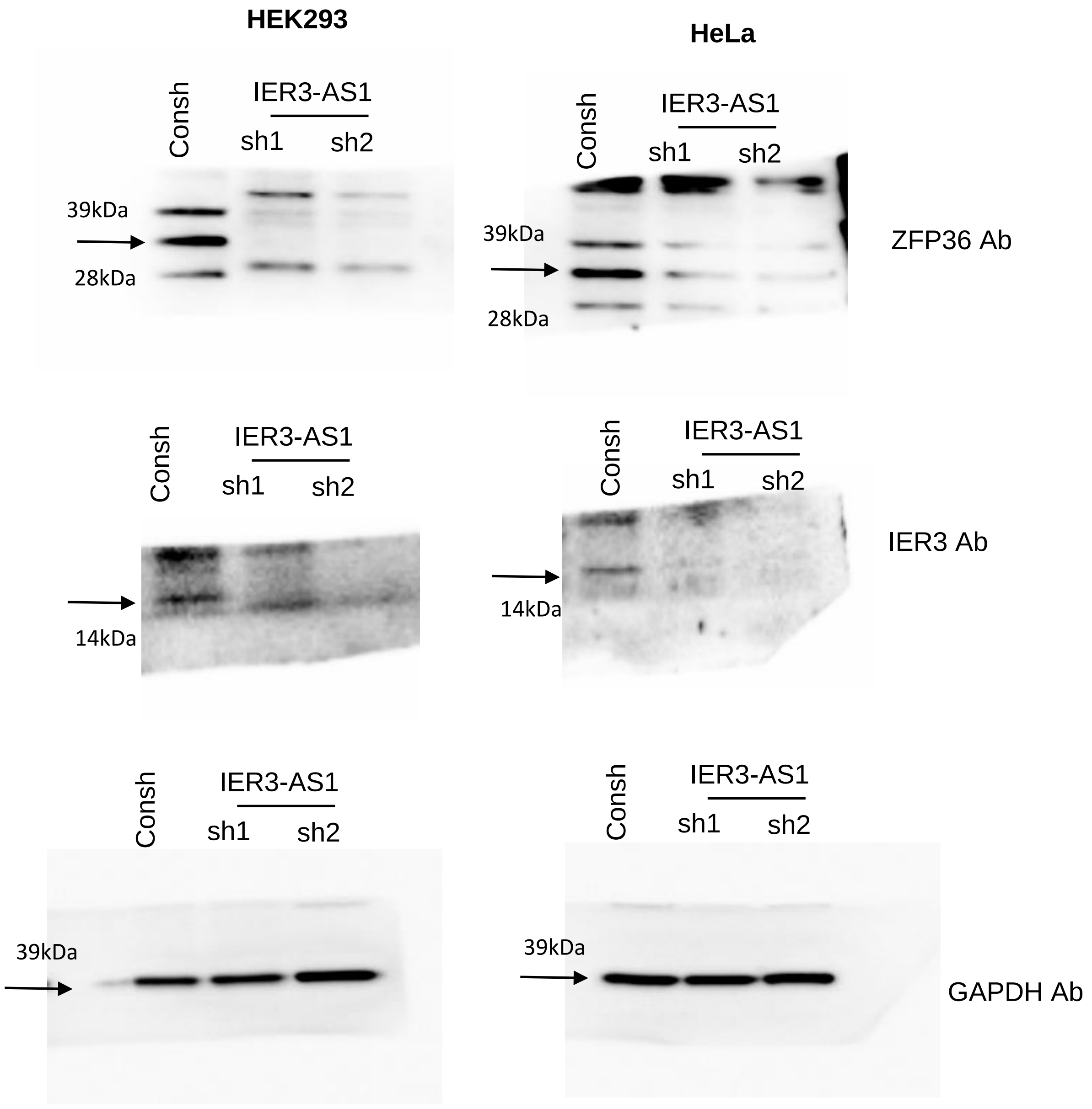

Supplementary Figure 5L

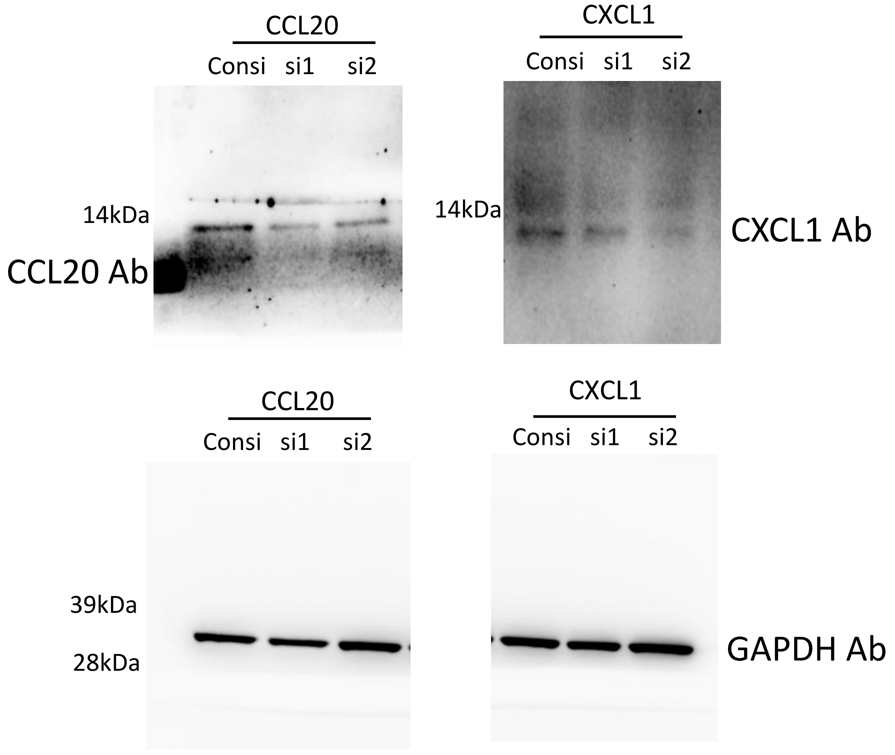

Supplementary Figure 5M

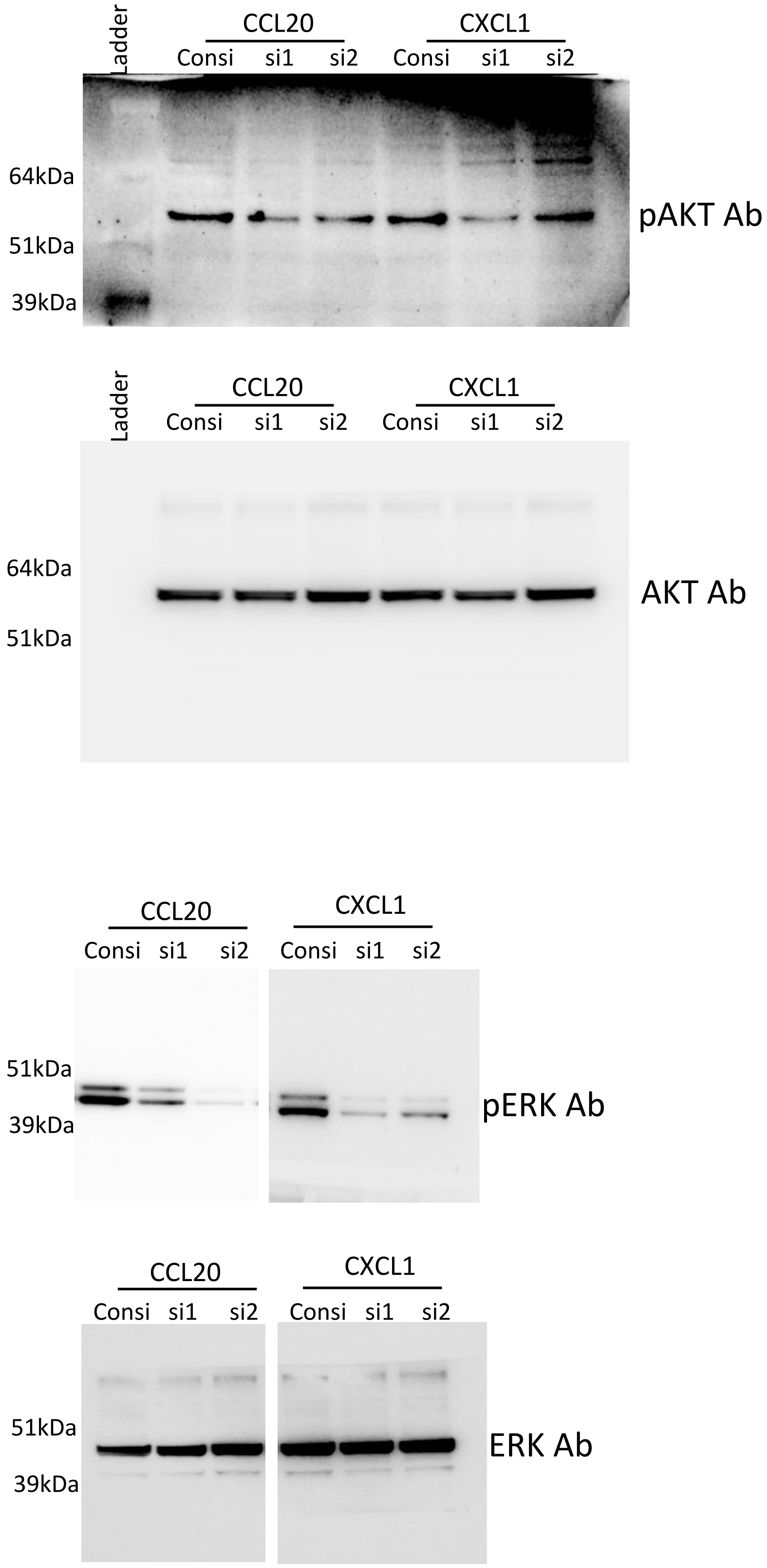

Supplement: Supplementary file 8 — Source Data [file 41467_2022_32537_MOESM8_ESM.zip › Source data folder/Supplementary source data file for western blots_Updated.pdf]
